# Syndesmos functions as a tumor suppressor by facilitating epithelial cell adhesion mediated by interactions of E-cadherin and β-catenin

**DOI:** 10.1038/s41419-026-08857-0

**Published:** 2026-05-19

**Authors:** Jisun Hwang, Ga-Eun Lim, Bohee Jang, Jee Young Sung, Hyewon Shim, Hyeryun Kwon, Eun Kyung Hong, Eek-hoon Jho, Yong-Nyun Kim, Eok-Soo Oh

**Affiliations:** 1 Department of Life Sciences, Ewha Womans University, Seoul, South Korea; 2 Cancer Metastasis Branch, Division of Cancer Biology, National Cancer Center, Gyeonggi-do, Goyang-si, South Korea; 3 Department of Life Sciences, University of Seoul, Seoul, South Korea; 4 Department of Pathology, National Cancer Center, Gyeonggi-do, Goyang-si, South Korea

**Keywords:** Colon cancer, Epithelial-mesenchymal transition

## Abstract

Syndesmos (SDOS), which regulates cytoskeletal organization via interaction with syndecan-4 in fibroblasts, was under-expressed in highly metastatic HCT116 colon cancer cells compared to weakly metastatic HT29 cells. SDOS overexpression decreased migration and proliferation of HCT116 cells, while siRNA-mediated knockdown of SDOS increased these activities in HT29 cells. SDOS overexpression in HCT116 cells promoted epithelial cell-like morphology, enhanced cell-cell and cell-ECM junction formation, and increased expression of epithelial markers. SDOS also elevated adhesion receptors, such as E-cadherin and syndecan-4, and their intracellular anchoring proteins. Notably, SDOS specifically interacted with E-cadherin and β-catenin, enhancing their interaction at cell-cell junctions in both HCT-SDOS and HT29 cells, suggesting a direct role of SDOS in adherens junction formation. In colon cancer tissue samples, SDOS expression was lower compared to adjacent normal tissues and lower SDOS levels were associated with poor post-progression survival in patients. In vivo, SDOS overexpression inhibited tumor growth in a HCT116 xenograft mouse model. Silencing SDOS in CT26-luc cells via siRNA increased tumor growth in the cecum and liver metastasis in an orthotopic model. These findings suggest that SDOS plays a critical role in suppressing colon cancer development and metastasis by maintaining epithelial architecture and promoting cell-cell and cell-ECM adhesion.

## Introduction

Syndesmos (SDOS), also known as NUDT16L1 or TIRR [1, 2], was initially identified through a yeast two-hybrid screen for intracellular proteins that interact with the cytoplasmic domain of syndecan-4 in focal adhesions [3]. SDOS promotes focal adhesion formation, actin stress fiber formation, and cell spreading in NIH 3T3 cells, and these processes depend on its interaction with syndecan-4 [3]. SDOS also binds to other focal adhesion adaptor proteins, such as paxillin and Hic-5, to further enhance focal adhesion and actin stress fiber formation [4]. These findings suggest that SDOS plays a scaffolding role in cytoskeletal organization. The cytoskeleton serves as the fundamental framework of the cell; it spatially organizes subcellular components and comprises a dynamic network of filaments extending from the cytoplasm to the nucleus [5–8]. These structures provide the cellular architecture that supports various cellular functions across different cellular compartments [9]. In addition to focal adhesions, cell-cell junctions such as adherens junctions are critical for maintaining epithelial integrity. In normal epithelial cells, adhesion complexes formed by E-cadherin, a calcium-dependent cell-cell adhesion protein, are essential for maintaining tissue integrity and the structural cohesion of the cell layer [10–12]. This structural integrity is crucial for epithelial cell functions, including the barrier function between external and internal environments, the distinct tissue polarity of apical and basal surfaces, and tissue development and differentiation [13–15].

Epithelial cell architecture is mediated by the complex formed by E-cadherin and β-catenin, which is a cytosolic adaptor protein that connects to the cytoskeleton. In addition to ensuring cytoskeletal organization and tissue architecture, this complex also regulates various cellular signaling pathways [16, 17]. Dysfunction or abnormal expression of E-cadherin leads to aberrant morphological changes and abnormal functions, and thereby contributes to various diseases, including cancer [18–23]. The loss of E-cadherin expression disrupts cytoskeletal organization and subsequently induces cells to change from epithelial to mesenchymal morphology, in a process known as the epithelial-to-mesenchymal transition (EMT). This key process in epithelial cancer progression is characterized by reduced cell polarization and increased invasiveness [24, 25]. The loss of E-cadherin expression also results in the cytosolic accumulation of β-catenin, which translocates to the nucleus and interacts with TCF family members to regulate the expression levels of target genes, including cell cycle regulatory proteins [26]. This dysregulated gene expression promotes uncontrolled cell growth, a well-known phenomenon in cancer cells. Therefore, maintaining normal cell-cell and cell-ECM adhesion could serve as an important regulatory mechanism against carcinogenesis. Given that SDOS interacts with the cytoplasmic domain of syndecan-4 to regulate cytoskeletal organization in cell-ECM adhesion, it is plausible that SDOS also associates with other structural proteins to maintain epithelial cell architecture. However, the function of SDOS in epithelial cells remains largely unknown. Therefore, we investigated the role of SDOS as a potential tumor suppressor in colon cancer, one of the most common malignancies originating from epithelial tissues. Here, we examined the roles of SDOS in regulating cell-cell and cell-ECM adhesion, key processes that govern cancer cell behavior in colon cancer.

## Materials and methods

### Antibodies

The antibody against SDOS (NUDT16L1) was purchased from Abcam (ab154808, Cambridge, UK) and SIGMA- Aldrich (HPA044186 and SAB106808, St. Louis, MO, USA). Anti-paxillin antibody was obtained from Millipore (05-417, Billerica, MA, USA). The antibody against N-cad (sc-7939), cytokeratin18 (sc-32722), slug (sc-166476), TCF4 (sc-8631), as well as GAPDH (sc-47724) purchased from Santa Cruz Biotechnology (Santa Cruz, CA, USA). The antibodies against HA (#2367), Snail (#3895), LRP6 (#3395), phospho-LRP6 (#2568), LRP5 (#5540), FZD5 (#5266), Axin1 (#2087), phospho-GSK3 (#9336), TCF1/7 (#2206), DVL2 (#3216), and DVL3 (#3218) were purchased from Cell Signaling Technology (Danvers, MA, USA). The antibody against vinculin (V9131), β-actin (A5441) was purchased from SIGMA. Anti-E-cad antibody was purchased from Santa Cruz Biotechnology (sc-71007, Santa Cruz, CA, USA) and Cell Signaling Technology (#3195, Danvers, MA, USA). Anti-Vimentin antibody was purchased from GeneTex (GTX629743, Irvine, CA, USA) and SIGMA-Aldrich (V2258, St. Louis, MO, USA). The antibody against ZO-1 (339100), ZO-3 (36-4100), CLDN7 (34-9100) as well as FITC-phalloidin (F432) were purchased from ThermoFisher scientific (Waltham, MA, USA). Anti-GSK3β (610201) and anti-β-catenin (61054) antibodies were purchased from BD Transduction Laboratories (San Diego, CA, USA). The antibody against LEF1 (A303-487A) was purchased from Bethyl Laboratories (Montgomery, TX, USA).

### Plasmid construct and siRNA

The SDOS were inserted into the pcDNA3.1 expression vector (Invitrogen, MA, USA). Small interfering RNA (siRNA) against human SDOS (siRNA ID. 84309-1, -2, -3) was purchased from Bioneer Corp. (Daejeon, South Korea).

### Cell culture and transfection

The human colon cancer cell line HCT116 and HT29 were maintained in McCoy’s 5A medium (Hyclone, Cytiva) containing with 10% (v/v) fetal bovine serum (FBS, Hyclone) and gentamicin (50 μg/ml) (Sigma-Aldrich, St. Louis MO, USA). Those cells were cultured at 37 °C in a humidified 5% CO_2_ atmosphere. Each cell line was passaged fewer than 6 months, authenticated and tested for mycoplasma contamination prior to use. Mycoplasma test was performed using TaKaRa PCR Mycoplasma Detection Set (#6601, Takara Bio, Japan). Transient transfections were carried out using the VivaMagic transfection reagent (Vivagen Co., Gyeonggi-Do, South Korea) and siRNA transfection was performed in 60-mm dishes using Lipofectamine 2000 (Thermo Fisher Scientific, Waltham, MA, USA) reagents as described in the provided manual. To generated cell lines stably expressing the SDOS, HCT116 cells at 70% confluency were transfected with 1 μg of SDOS-pcDNA 3.1 (SDOS) or empty pcDNA3.1 (VEC) vector using VivaMagic and then selected in medium containing 500 μg/ml of G418 (EMD Biosciences, CA, USA) for 4 weeks. The surviving clones were individually isolated and analyzed by RT-PCR and immunoblotting.

### Cell proliferation assay

Cell proliferation was measured by a colorimetric assay using MTT (3-4,5-dimethylthiazolyl-2,5-diphenyltetrazolium bromide) according to the manual. Briefly, HCT116 (4 × 10^3^ cells/well), HCT116 cells stably expressing SDOS (2.5 × 10^3^ cells/well), and HT29 (6 × 10^3^ cells/well) cells were seeded to 96-well plates. Cells were allowed to attach to the plate for 24 h, medium containing 0.5 mg/ml MTT was added to each well, and cells were incubated for 1 h. The medium was then removed, and dimethyl sulfoxide (DMSO) was added to each well. The mean absorbance at 570 nm was measured using a 96-well micro plate reader (Molecular Devices, CA, USA).

### Anchorage-independent growth assay

The 6-well culture plates were coated with 3 ml/well bottom agar mixture (McCoy’s 5A, 10% FBS, 0.6% agar). After the bottom layer had solidified, 1 ml of top agar mixture (McCoy’s 5A, 10% FBS, 0.3% agar) containing either HCT116 cells (1 × 10^5^ cells/well) or HT29 cells (1 × 10^5^ cells /well) was added to each well, and the cultured were incubated at 37 °C in a humidified 5% CO_2_ atmosphere. Regular growth medium was gently layered over the cultures every 5 days. Colony formation was monitored daily with a light microscope. After 14 days, colonies were stained with 0.005% crystal violet and counted.

### Transwell migration assay

The lower surface of each Transwell insert (Costar, Cambridge, MA) was coated with gelatin (10 μg/ml) and the membranes were allowed to dry for 1 h at room temperature. The Transwell inserts were assembled to a 24-well plate (Costar, Cambridge, MA), and the lower chamber was filled with DMEM containing 20% FBS. HCT116 cells (2.5 × 10^5^) or HT29 cells (2 × 10^5^) were added to each upper chamber, and the plate was incubated at 37 °C in a 5% CO_2_ incubator for 24 h. Cells that migrated to the lower surface were fixed and stained with 0.6% hematoxylin and 0.5% eosin, and counted.

### Inverted centrifugal detachment assay

The 12-well culture plate were coated with 10 μg/ml of collagen or fibronectin for 1 h at 37 °C incubators. The plates were then washed with PBS and blocked with 0.2% BSA for 1 h at room temperature. Cells (1 × 10^6^ cells/well) were plated with serum-free medium and the cells were incubated for 2 h at 37 °C in a 5% CO_2_ incubator. The detached cells were removed with PBS, and the plates were filled with PBS, sealed with Parafilm, and centrifuged at 300 × *g* for 5 min using a tabletop plate centrifuge (LABOGENE, South Korea). After centrifugation, cells that attached to the plate were fixed with 100% methanol for 15 min, and stained with crystal violet solution (0.1% crystal violet and 10% ethanol). The medium was removed and tried, and the stained cells were eluted with elution buffer (5% acetic acid and 5% methanol). The mean absorbance at 595 nm was measured using a 96-well plate reader (Molecular Devices, CA, USA).

### Cell attachment assay

Cell attachment to ECM molecules was monitored in real time using an xCELL-ignece system (Roche Diagnostics GmbH, Basel, Switzerland). For cell spreading, E-plate 16 assemblies (ACEA Biosciences, Santa Clara, CA, USA) were coated with 10 μg/ml of fibronectin or collagen at 37 °C for 1 h. Cells were seeded to each well and incubated at 37 °C for 15 min. The plate was assembled on the RTCA DP Analyzer (RTCA software version 1.2, ACEA Biosciences), and the cell adhesion was monitored at 2-min intervals for 90 min at 37 °C. The obtained data were analyzed using the provide RTCA software.

### RT-PCR

Total RNA from cells was extracted with easy-BLUE^TM^ (iNtRON Biotechnology, Seongnam-si, Gyeonggi, KOREA) and then 3 µg of RNA were used as template for reverse transcriptase reaction. PCR was performed using specific primers of Mouse SDOS, (forward) 5’-TAC GCC TTA CCG AAG CTG AT-3’ and (reverse) 5’-TGT GTA CAG TGG GAC CCG TA-3’; Human SDOS, (forward) 5’-GAC TAC CTG AGC TCG CAC CT-3’ and (reverse) 5’- ACT CGG TCC TTC TGG GTG TA-3’; Human SDC4, (forward) 5’-TGGGGGCTTTCTTGTAGATG-3’ and (reverse) 5’- GTCTGGCTCTGGAGATCTGG-3’; Human CDH1 (E-cadherin), (forward) 5’-TCATGAGTGTCCCCCGGTAT-3’ and (reverse) 5’-TCTTGAAGCGATTGCCCCAT-3’; Human CDH2 (N-cadherin), (forward) 5’-TGACACTGTGGAGCCTGAT-3’ and (reverse) 5’-GTGGAGCCACTGCCTTCATA-3’; Human SNAI1 (SNAIL), (forward) 5’- TTCCAGCAGCCCTACGACCAG-3’ and (reverse) 5’-CGGACTCTTGGTGCTTGTGGA -3’; Human SNAI2 (SLUG), (forward) 5’-AAGCATTTCAACGCCTCCAA-3’ and (reverse) 5’- AAGGTAATGTGTGGGTCCGA -3’; Human TWIST, (forward) 5’-GGAGTCCGCAGTCTTACGAG -3’ and (reverse) 5’-TCTGGAGGACCTGGTAGAGG-3’; Human CTNNB1 (beta-catenin), (forward) 5’-TCCCACTAATGTCCAGCGTT-3’ and (reverse) 5’- AAGCATTTTCACCAGGGCAG-3’; Human Tjp1 (forward) 5’-ACAGGTGTACAGGAAGGAGC-‘ and (reverse) 5’-GGGATGTTGTCTGGAGTCCA-3’; Human Tjp3, (forward) 5’-GGTACCAGGAAGTCAAGGCT-3’ and (reverse) 5’-TCAGCCTGTGTCTCCCATTT-3’; Human Cldn7, (forward) 5’-ACTGCTGGGCTTTTCAATGG-3’, (reverse) 5’-CAGGAACATGCTACCAAGGC-3’ Human GAPDH, (forward) 5’-CCACCCATGGCAAATTCCATGGCA-3’ and (reverse) 5’-TCTAGACGGCAGGTCAGGTCCACC-3’; After an initial denaturation at 94 °C for 5 min, the samples were subjected to 30 cycles of denaturation at 94 °C for 30 s, annealing at 55 °C for 1 min, extension at 72 °C for 1 min. The PCR products were separated by 1% agarose gel electrophoresis.

### Immunoblotting

Cells were washed twice with PBS and lysed in RIPA buffer (50 mM Tris, pH 8.0, 150 mM NaCl, 1% Nonidet P-40) containing SDS (0.5 or 2.5%) to obtain whole-cell lysates. The lysis buffer contained protease inhibitors (1 µg/ml aprotinin, 1 µg/ml antipain, 5 µg/ml leupeptin, 1 µg/ml pepstatin A, and 20 µg/ml phenylmethylsulfonyl fluoride) and phosphatase inhibitors (10 µM NaF and 2 µM Na_3_VO_4_). Cell lysates were incubated on ice for 20 min and clarified by centrifugation at 13,000 rpm for 15 min at 4 °C. Lysates were then boiled for 5 min in SDS sample loading buffer. For detection of Frizzled 5, NuPAGE™ LDS Sample Buffer (4×; Thermo Fisher Scientific, Waltham, MA, USA) and 10× dithiothreitol (DTT) were used instead of SDS sample loading buffer, and samples were incubated at 70 °C for 10 min. Prepared protein samples were separated by 8%, 10%, or 12% SDS-PAGE, transferred to nitrocellulose membranes (GE Healthcare, Chicago, IL, USA) or PVDF membranes (ATTO, Motoasakusa, Tokyo, Japan), and probed with the indicated antibodies. Signals were detected using the Odyssey CLx imager (LI-COR Biosciences, Lincoln, NE, USA) and analyzed with Image Studio Lite software (LI-COR Biosciences). Signals of Wnt signaling proteins were detected using an enhanced chemiluminescence kit (ELPIS, Millipore, Thermo Scientific) and the MicroChemi 4.2 system (DNR Bio-Imaging Systems, CA, USA).

### RNA sequencing analysis

Total RNAs from HCT116-SDOS cells were extracted using TRIzol^TM^ reagent (Invitrogen, Waltham, MA, USA). The cDNA library construction, library purification and transcriptome sequencing were performed at Macrogen (Seoul, South Korea).

### Immunofluorescence

Cells on coverslips in a 12-well plate were fixed with 3.5% paraformaldehyde for 10 min, washed with PBS, permeabilized with 0.5% Triton X-100 in PBS for 10 min, blocked with 0.5% BSA, and then incubated overnight with the appropriate antibodies. After cells were incubated with fluorescent dye-conjugated secondary antibodies (Thermo Fisher Scientific) for 1 h, coverslips were mounted on microscope slides with mounting medium containing DAPI (Vector Laboratories, Burlingame, CA, USA). The fluorescence images were visualized under a confocal microscope a Zeiss LSM880 Airyscan (Carl Zeiss, Inc., Oberkochen, Germany) at Ewha Fluorescence Core Imaging Center and analyzed using ZEN software (Carl Zeiss).

### Proximity ligation assay

Cells were grown in 24-well plates into glass coverslips. Cells were fixed with 3.5% paraformaldehyde for 10 min and permeabilized using 0.5% Triton X-100 for 10 min. The next steps were performed according to manufacturer’s instructions (NaveniFlexTM Cell MR, Navinci). The coverslips were mounted with mounting medium containing DAPI (Vector Laboratories, Burlingame, CA, USA). The fluorescence images were visualized under a confocal microscope (Carl Zeiss, Oberkochen, Germany) and analyzed using ZEN software (Carl Zeiss).

### Tissue array

Colorectal cancer tissue arrays were purchased from Superbiochips Laboratories (Seoul, Korea) as previously described [27]. Each array contained 60 sections collected from 60 patients by biopsy or surgical resection. The tumor tissues were incubated with primary antibodies for 1 h and then treated with biotinylated anti-rabbit antibody (1:1000 dilutions; Vector Laboratories) for 1 h. The color reaction was developed by incubation with diaminobenzidine solution (Sigma, St. Louis, MO) and subsequent counterstaining with hematoxylin. Parallel sections incubated with normal IgG instead of primary antibodies were used as negative controls. Overall staining results were scored from 0 to 3 based on intensity and positive staining rate. The intensity of staining was categorized as 0, negative (−); 1, weak (+); 2, intermediate (++). The stained tissue arrays were reviewed by experienced pathologists.

### Survival analysis

Colon cancer survival datasets from KM-Plotter (http://kmplot.com/analysis) were analyzed to assess survival differences. The analysis settings included all samples and stratification by tumor stage. For stage-specific analysis, tumor stages were grouped into early-stage (stage 1 + 2) and advanced-stage (stage 3 + 4). Kaplan–Meier survival curves were generated for each group, and statistical differences were assessed using the log-rank test.

### Animal models and ethical approval

For the xenograft mouse model, HCT116 cells were stably transfected with empty vector (VEC) or vector encoding SDOS (SDOS) (1 × 10^6^ cells/mouse) and cells were subcutaneously injected below the dorsal flank into 5-week-old male BALB/c nude mice (*n* = 5 per group; Orient Bio Co., Seongnam, Korea). Tumor growth was monitored every 3 days from day 10 post-injection using calipers, and tumor volume was calculated using the following formula: volume = (Length × Width^2^)/2. For the orthotopic mouse model, CT26-luc cells (1 × 10^6^ cells/mouse) transfected with either si-NC or si-SDOS were injected into the cecum of 6-week-old female BALB/c mice (*n* = 6 per group; Orient Bio Co., Seongnam, Korea). From 2 weeks post-injection, in vivo tumor growth was monitored weekly until day 42 using an IVIS Imaging System (IVIS Lumina XRMS, Revvity, Waltham, MA, USA). Prior to bioluminascence imaging, mice were intraperitoneally injected with D-luciferin (150 mg/kg) for 10 min and anesthetized with 1% isoflurane. On day 43, mice were re-administered D-luciferin, sacrificed, and the cecal tumors and livers were resected for ex vivo IVIS imaging. The animal study protocols were approved by the Institutional Animal Care and Use Committee of National Cancer Center (Approval #NCC-23-890, #NCC-24-1007) and conducted in accordance with ARRIVE guidelines [28]. Sample sizes were selected based on pilot experiments and previous studies, ensuring they are consistent with standard practices for similar xenograft and orthotopic tumor models in the field [29, 30]. In this study, formal randomization was not performed for group allocation as the experimental groups were inherently determined by the specific cell types or transfection conditions injected into the mice. To minimize potential bias, tumor growth was monitored and analyzed in a blinded manner, and investigators remained blinded to group allocation during outcome assessment. In accordance with IACUC guidelines, tumor size did not exceed 2 cm in diameter during the animal study.

### Immunohistochemistry

Tumor tissues were fixed with 10% neutral buffered formalin, paraffin-embedded, and sectioned at a thickness of 4 μm. Sections were dried at 56 °C for 1 h, and subjected to immunohistochemical staining using a Discovery XT automated staining system (Ventana Medical Systems, Tucson, AZ, USA). Sections were deparaffinized and rehydrated using EZ Prep solution (Ventana Medical Systems), followed by washing with reaction buffer. Antigen retrieval was performed by heat treatment using Tris-EDTA buffer (pH 8.0) or citrate buffer (pH 6.0), as appropriate, at 90 °C for 30 min. Sections were incubated with primary antibodies against SDOS, E-cad, N-cad, vimentin, followed by the appropriate secondary antibodies. After immunostaining, sections were counterstained, dehydrated, and mounted. Stained slides were scanned using a VS200 slider scanner (Olympus, Tokyo, Japan).

### Statistical analysis

Experiments and data analysis were performed in a blinded manner. Data are represented as the mean ± standard deviation (SD) or as the mean ± standard error of the mean (SEM) from at least three independent experiments. Sample sizes as well as the definitions of statistical methods and measures for each experiment are provided in the corresponding figure legend. Statistical analysis was performed using an unpaired two-tailed Student’s *t* test or two-way analysis of variance (ANOVA) followed by Sidak’s multiple comparisons test, as appropriate. A *p* value less than 0.05, 0.01, and 0.001 was considered statistically significant.

## Results

### SDOS expression negatively regulates the tumorigenic activities of colon cancer cells

**Fig. 1:**
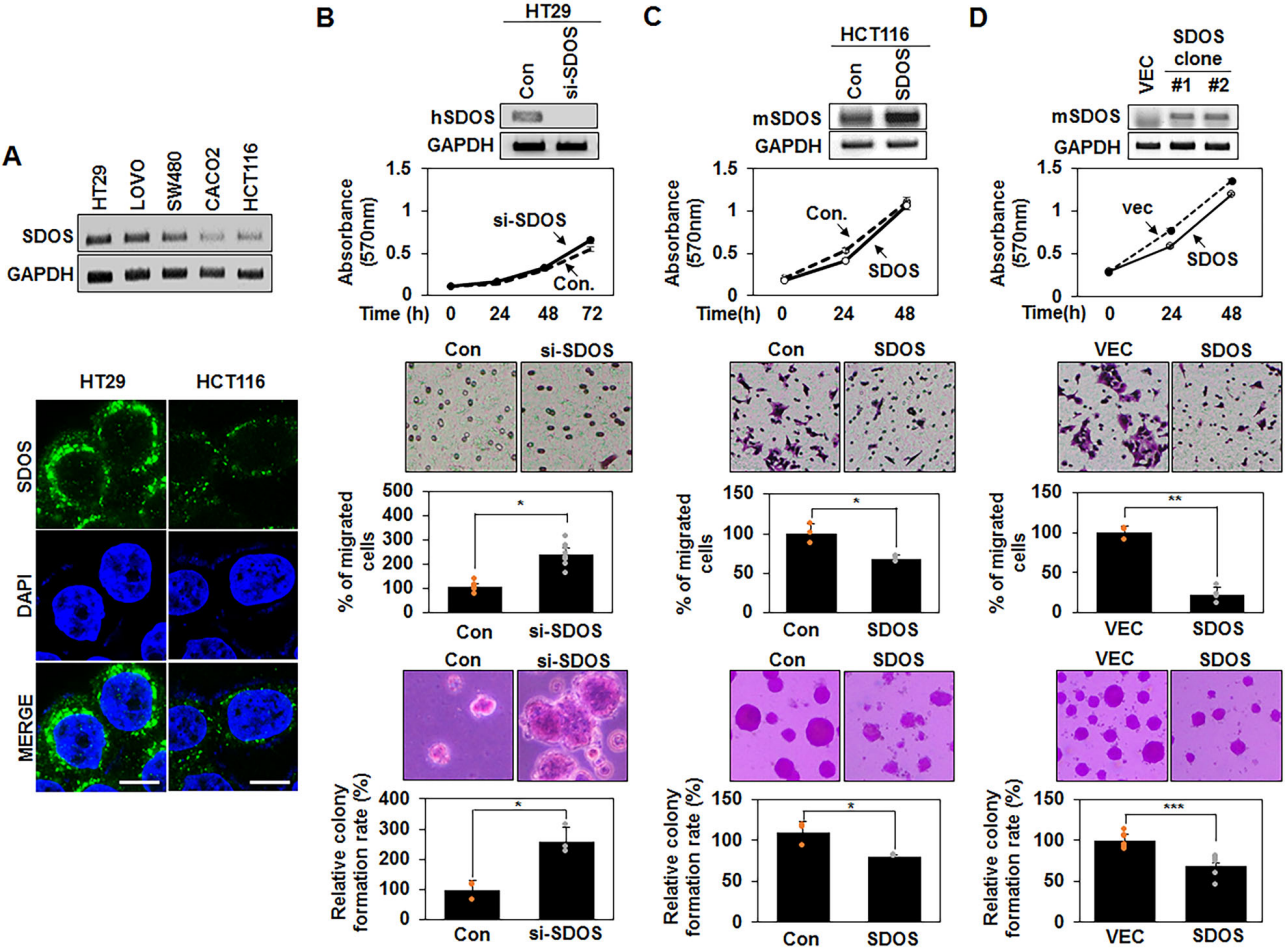
SDOS expression negatively regulates the tumorigenic activities of colon cancer cells. **A** Basal mRNA expression levels of SDOS in the indicated colon cancer cell lines were analyzed by RT-PCR, with GAPDH used as the loading control (top). Representative confocal immunofluorescence staining images of SDOS are shown, with nuclei counterstained with DAPI for visualization (bottom). HT29 cells were transfected with siRNA against SDOS (**B**) or HCT116 cells were transiently transfected with mouse SDOS (**C**). mRNA expression levels of SDOS were analyzed by RT-PCR and cell viability was determined by MTT assay (top). Transwell migration assay (middle) and colony formation assay (bottom) were performed as described in the Experimental Procedures. **D** HCT116 cells were stably transfected with either empty vector (VEC) or vector encoding the mouse SDOS (SDOS). mRNA expression of SDOS was analyzed by RT-PCR (top). MTT assay (middle) and cell migration assay (bottom) were performed. Results are representative of three independent experiments. Data are presented as mean ± standard deviation (SD). Significant differences between groups are denoted as ^*^p < 0.05, ^**^p < 0.01, ^***^p < 0.001.

To investigate the relationship between SDOS expression and tumor suppression, we first analyzed the mRNA expression levels of SDOS in various colon cancer cell lines, including weakly metastatic cells (HT29, LOVO, and SW480) and highly metastatic cells (CACO2 and HCT116, Fig. 1). Interestingly, the SDOS mRNA was more highly expressed in weakly metastatic HT29, LOVO, and SW480 cells compared to highly metastatic CACO2 and HCT116 cells (Fig. 1A, top). Consistently, immunofluorescence analysis revealed that SDOS expression was much lower in HCT116 cells compared to HT29 cells (Fig. 1A, bottom), suggesting that SDOS may negatively regulate the tumorigenic activities of colon cancer cells. Consistent with this notion, specific siRNA-mediated SDOS knockdown resulted in significantly increased migration and anchorage-independent growth of HT29 cells, but not proliferation, compared with control siRNA-transfected cells (Fig. 1B). Conversely, HCT116 cells with transient (Fig. 1C) or stable (Fig. 1D) expression of exogenous SDOS showed reduced migration and anchorage-independent growth. These results suggest that SDOS expression downregulates the tumorigenic activity of colon carcinoma cells.

### SDOS expression induces cell adhesion-related genes in HCT116 cells

**Fig. 2:**
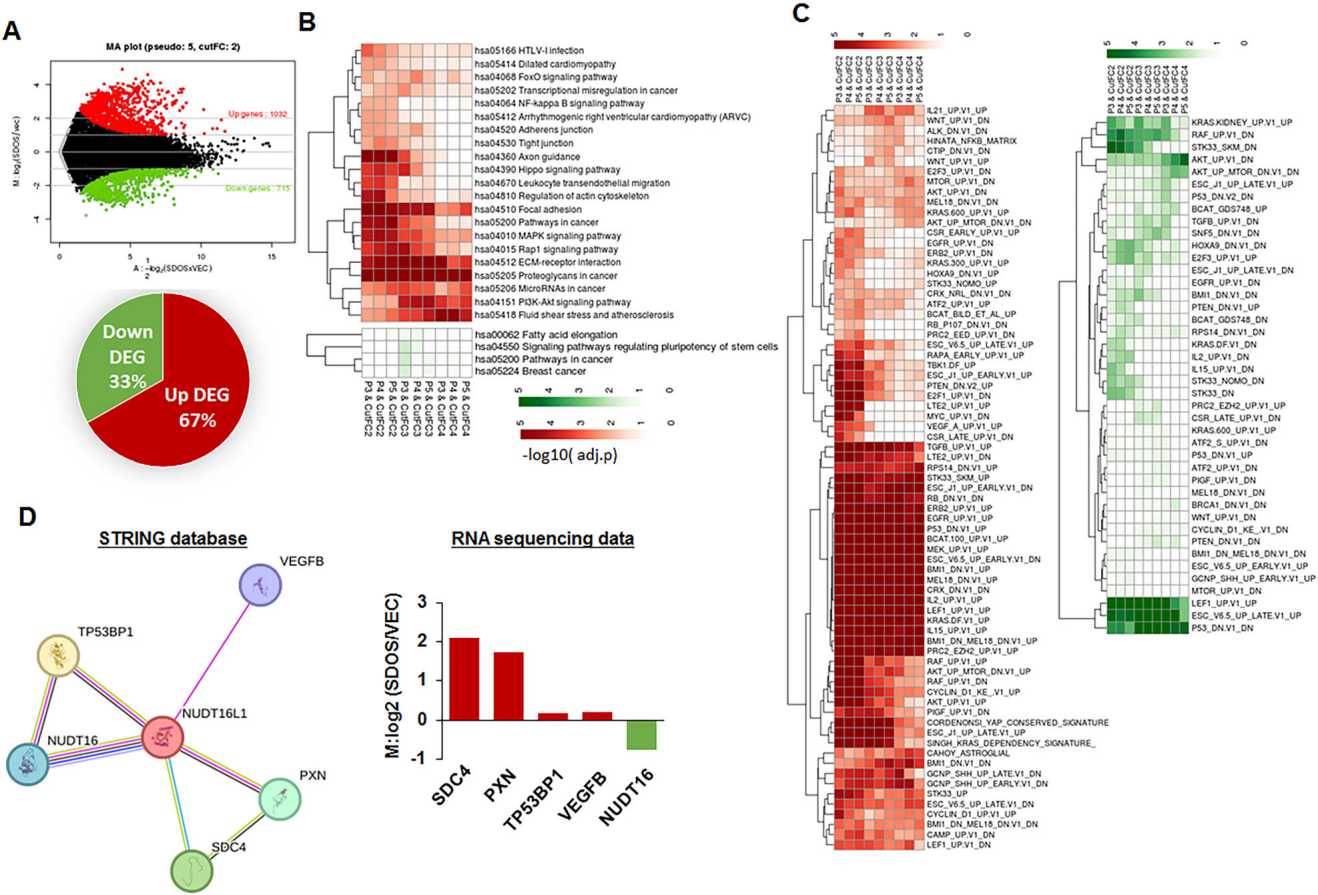
Differentially expressed gene levels in HCT-SDOS cells. **A** The MA plot from DEGs represents up-regulated genes (red dots) and down-regulated genes (green dots) in HCT-SDOS cells compared to levels in vector cells (top) and Pie chart represents the percentage of up-regulated and down-regulated genes in HCT-SDOS cells (bottom). **B** KEGG enrichment analysis for DEGs are shown. **C** MSigDB pathway enrichment analysis for DEGs. **D** Computational prediction of SDOS protein binding proteins network using STRING database (left). mRNA expression levels of encoding SDOS binding proteins (right).

To further examine how SDOS downregulates the tumorigenic activities of cancer cells, we used RNA-seq analysis to identify differentially expressed genes (DEGs) in HCT116 cells stably expressing SDOS (HCT-SDOS) compared to vector control cells (Fig. 2). Biologically meaningful genes were selected by a log_2_ fold change >2 and a pseudo count > 5 (Fig. 2A, top). From a total human genes screened, we identified significant differences in 1747 genes; of them, 1032 were upregulated (67%) and 715 were downregulated (33%) (Fig. 2A, bottom). Kyoto Encyclopedia of Genes and Genomes (KEGG) analysis showed that the genes differentially expressed in HCT-SDOS cells compared to vector control cells were enriched in 25 pathways, particularly those involving proteoglycans in cancer, ECM-receptor interactions, focal adhesions, adherens junctions, and tight junctions (Fig. 2B). Interestingly, analysis of the MSigDB pathway associated with C6 oncogenic signature genes revealed that HCT-SDOS cells were enriched for various tumor suppressor genes, including those related to cell adhesion (e.g., CDH1, EPCAM, FAT1, and SPRED1), Wnt signaling (e.g., RNF43, AXIN2, and TCF7L2), and the regulation of ECM remodeling (e.g., ETV6) (Fig. 2C). The genes encoding the known SDOS-binding proteins, SDC4, TGFB1l1, and PXN, were identified in the binding protein network generated by the STRING database computational prediction program (Fig. 2D, left), supporting the reliability of our analysis. Moreover, the expression levels of these genes were higher in HCT-SDOS cells than in HCT116-VEC cells (Fig. 2D, right). Together, these data suggest that SDOS negatively regulates the tumorigenic activity by maintaining epithelial cell architecture, likely through regulating both cell-cell and cell-ECM interaction-associated aspects of cytoskeletal organization.

### SDOS expression regulates cell-ECM interactions via regulation of the actin cytoskeleton

**Fig. 3:**
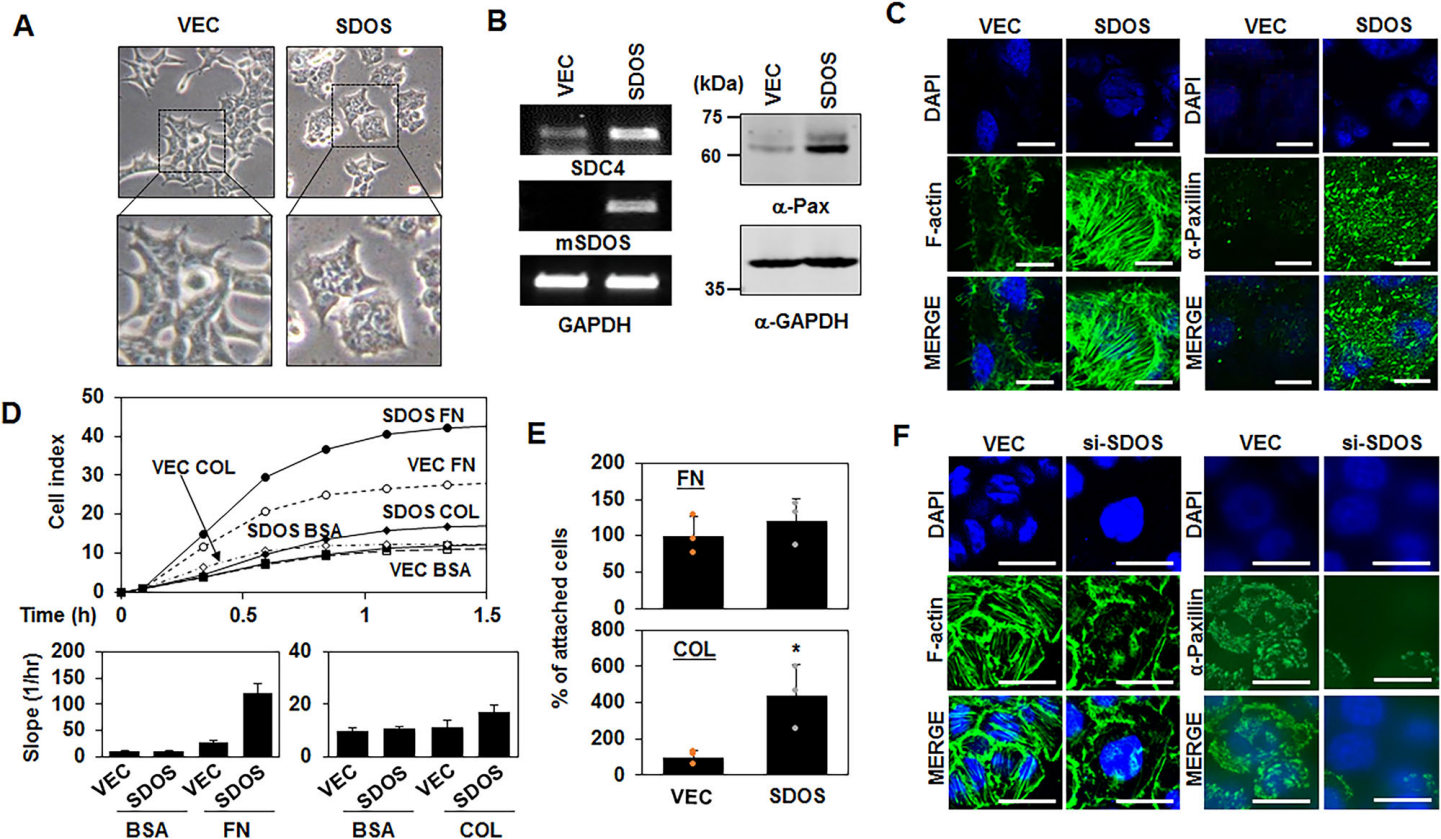
SDOS expression induces actin stress fiber and focal adhesion in colon cancer cells. **A**–**E** Control HCT116 cells (Vec) or HCT-SDOS cells (SDOS) were plated and incubated for 48 h. **A** Morphological changes were observed under bright field microscopy. **B** Total RNA was extracted, and mRNA expression was determined by RT-PCR (left). Paxillin levels were measured by Western blotting (right), with GAPDH as a loading control. **C** Representative confocal immunofluorescence images of F-actin (left) and paxillin (right) are shown. Nuclei were counterstained with DAPI. **D** Cell spreading on E-plates coated with fibronectin (FN) or collagen (COL) was monitored using the xCELLigence system. **E** Adhesiveness to the ECM was quantified using an inverted centrifugal detachment assay. **F** HT29 cells were transfected with siRNA targeting human SDOS (si-SDOS). Representative confocal immunofluorescence images of F-actin (left) and paxillin (right) are shown. Nuclei were counterstained with DAPI. Data are presented as mean ± standard deviation (SD). Significant differences between groups are denoted as ^*^p < 0.05.

Since cell architecture is tightly regulated by various cell junctions and the actin cytoskeleton, we next investigated the effect of SDOS expression on adhesion-mediated actin organization (Fig. 3). Consistent with the increased expression of genes involved in cytoskeletal organization (Fig. 2), HCT-SDOS cells exhibited a more epithelial-like cell morphology compared with control cells, including increased cell-cell contacts and a less spread out morphology (Fig. 3A). Consistent with a previous report [4], SDOS expression also increased the expression levels of syndecan-4 and paxillin (Fig. 3B). This suggests that the SDOS-mediated cytoskeletal organization of epithelial cells in the basal layer is regulated through focal adhesions. Indeed, SDOS expression increased the formation of actin stress fibers and focal adhesions in HCT-SDOS cells (Fig. 3C). Consistent with this increase of focal adhesion formation, a real-time cell spreading assay performed using the xCELLigence system showed that SDOS expression enhanced the spreading of HCT116 cells on both fibronectin and collagen (Fig. 3D). SDOS expression also increased the cell-substratum adhesion (i.e., cell-fibronectin and cell-collagen adhesiveness) of HCT116 cells, as quantified using an inverted centrifugal detachment assay (Fig. 3E). In contrast, paxillin staining showed that SDOS knockdown reduced the formation of actin stress fibers and focal adhesion formation in HT29 cells (Fig. 3F). Together these data suggest that loss of SDOS expression during cancer progression decreases cell-ECM interactions via reorganization of the cytoskeletal architecture and subsequent loss of tumor suppressor ability among colonic epithelial cells bearing cell-cell junctions.

### Exogenous expression of SDOS restores epithelial cell adhesion in colon cancer cells

**Fig. 4:**
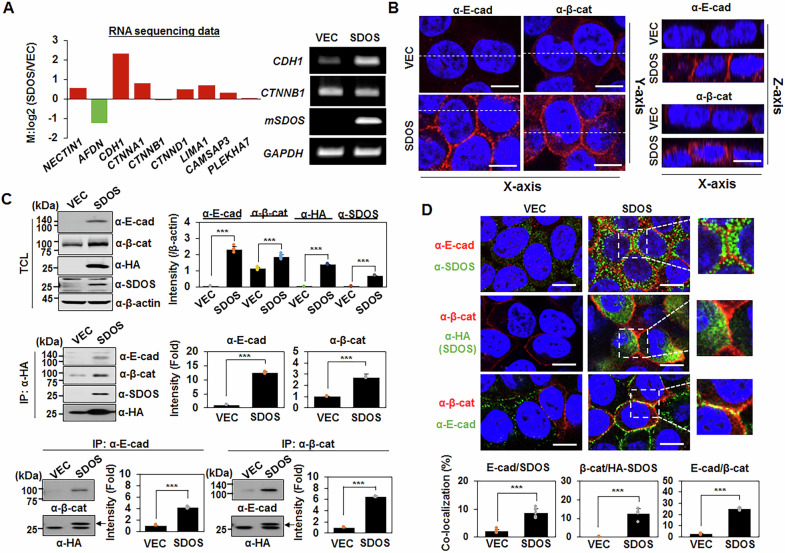
SDOS expression enhanced adherens junction formation in HCT116 cells. **A** Expression of adherens junction protein genes were analyzed by RNA sequencing (left). Total RNA was extracted and mRNA levels of CDH1 (E-cadherin) and CTNNB1 (β-catenin) were determined by RT-PCR (right). **B** The confocal immunofluorescence staining images of E-cadherin (E-cad, left), β-catenin (β-cat, middle), and the XZ view of cells were presented (right). **C** Expression levels of adherens junction proteins were measured by Western blotting (WB), with β-actin as a loading control (left). Total cell lysates (TCL) were incubated with anti-HA antibody to pull down the immune complexes and then subjected to Western blotted with anti-SDOS antibody (middle). Immune complexes precipitated with either anti-E-cad or -β-cat antibodies were subjected to WB with the indicated antibodies (bottom). Protein expression levels was quantified (right). **D** Representative confocal immunofluorescence staining images of E-cad and SDOS (top), β-cat and SDOS (middle), and β-cat and E-cad (bottom) are shown. The percentage of colocalization with proteins in the field was plotted (bottom). The nuclei were counterstained with DAPI. Data are presented as mean ± standard deviation (SD). Significant differences between groups are denoted as ^***^p < 0.001.

**Fig. 5:**
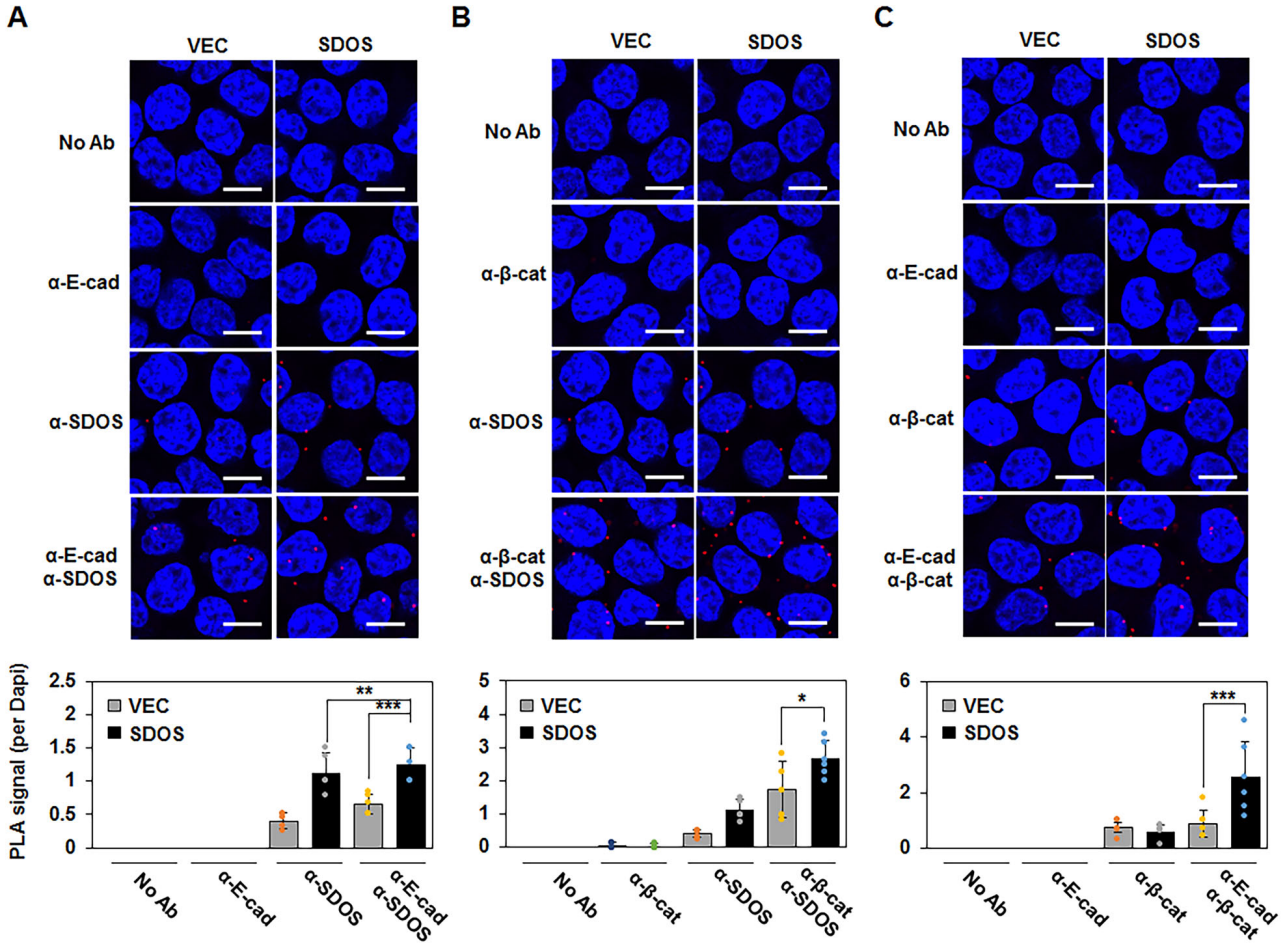
SDOS expression enhances the interaction E-cadherin and β-catenin in HCT116 cells. The in situ proximity ligation assay using HCT116 cells was performed with anti-E-cadherin (E-cad) and anti-SDOS antibodies (**A**), anti-β-catenin (β-cat, mouse) and anti-SDOS antibodies (**B**), anti-E-cad and anti-β-cat (rabbit) antibodies (**C**). Images of confocal immunofluorescence staining are shown (top). The nuclei were counterstained with DAPI. The PLA signals in the field were shown graphically (bottom). Data are presented as mean ± standard deviation (SD). Significant differences between groups are denoted as ^*^p < 0.05, ^**^p < 0.01, ^***^p < 0.001.

Since EMT involves dramatic changes in cell architecture and causes solid tumors to lose cell-cell interactions and become more malignant, we next investigated the cell-cell junction formation ability of HCT-SDOS cells. Regarding adherens junctions, which are important for the maintenance of cell-cell interactions, both RNA-seq and RT-PCR analyses showed significantly increased mRNA expression of E-cadherin, a critical component of adherens junctions in epithelial cells, compared with mock-transfected cells (Fig. 4A). Notably, SDOS overexpression increased the protein levels of E-cadherin and β-catenin, and staining with antibodies against E-cadherin and β-catenin revealed that these cells exhibited increased formation of adherens junctions, particularly in the apical aspect of HCT-SDOS cells (Fig. 4B). Furthermore, E-cadherin and β-catenin were detected in immunoprecipitates pulled down with anti-HA antibodies recognizing SDOS and immunoprecipitates with anti-E-cadherin or anti-β-catenin antibodies showed a significant enrichment of SDOS in HCT-SDOS cells compared to vector controls (Fig. 4C). Under our experimental conditions, SDOS colocalized with E-cadherin and β-catenin in ~8.6% and 12.5% of HCT-SDOS cells, respectively, whereas E-cadherin and β-catenin colocalized in 25% in these cells (Fig. 4D). A proximity ligation assay showed that SDOS interacted with E-cadherin and β-catenin in HCT-SDOS cells (Fig. 5A, B) and SDOS expression enhanced the interaction of E-cadherin with β-catenin (Fig. 5C). Consistently, in HT29 cells, which endogenously express a relatively high level of SDOS, endogenous SDOS interacted with E-cadherin and β-catenin at the cell surface (Supplementary Fig. S1A), was coimmunoprecipitated with both E-cadherin and β-catenin (Supplementary Fig. S1B), and colocalized with both E-cadherin and β-catenin at cell-cell junctions (Supplementary Fig. S1C). Together, these data suggest that SDOS enhances the formation of adherens junctions in epithelial cells, probably through interactions with E-cadherin and β-catenin. Since adherens junction formation is known to lead to assembly of tight junctions in epithelial cells [31–33], we further investigated the effect of SDOS expression on tight junction formation in HCT-SDOS cells (Supplementary Fig. S2A). Our RNA-seq (Supplementary Fig. S2A) and RT-PCR (Supplementary Fig. S2B) analyses showed increased mRNA expression of tight junction-related genes, including *Tjp1*, *Tjp3*, and *CLDN7*, in HCT-SDOS cells. Western blotting confirmed elevated protein levels of ZO-1, ZO-3, and claudin-7 upon SDOS overexpression (Supplementary Fig. S2C). Immunostaining revealed enhanced tight junction formation, as indicated by increased ZO-1 and ZO-3 staining, but not CLDN7 staining, in HCT-SDOS cells (Supplementary Fig. S2D). However, neither ZO-1 nor ZO-3 was detected in SDOS immunoprecipitates from HCT-SDOS cells (Supplementary Fig. S2E), suggesting that SDOS indirectly promotes tight junction formation by stabilizing adherens junctions.

### Exogenous expression of SDOS inhibits EMT in colon cancer cells

**Fig. 6:**
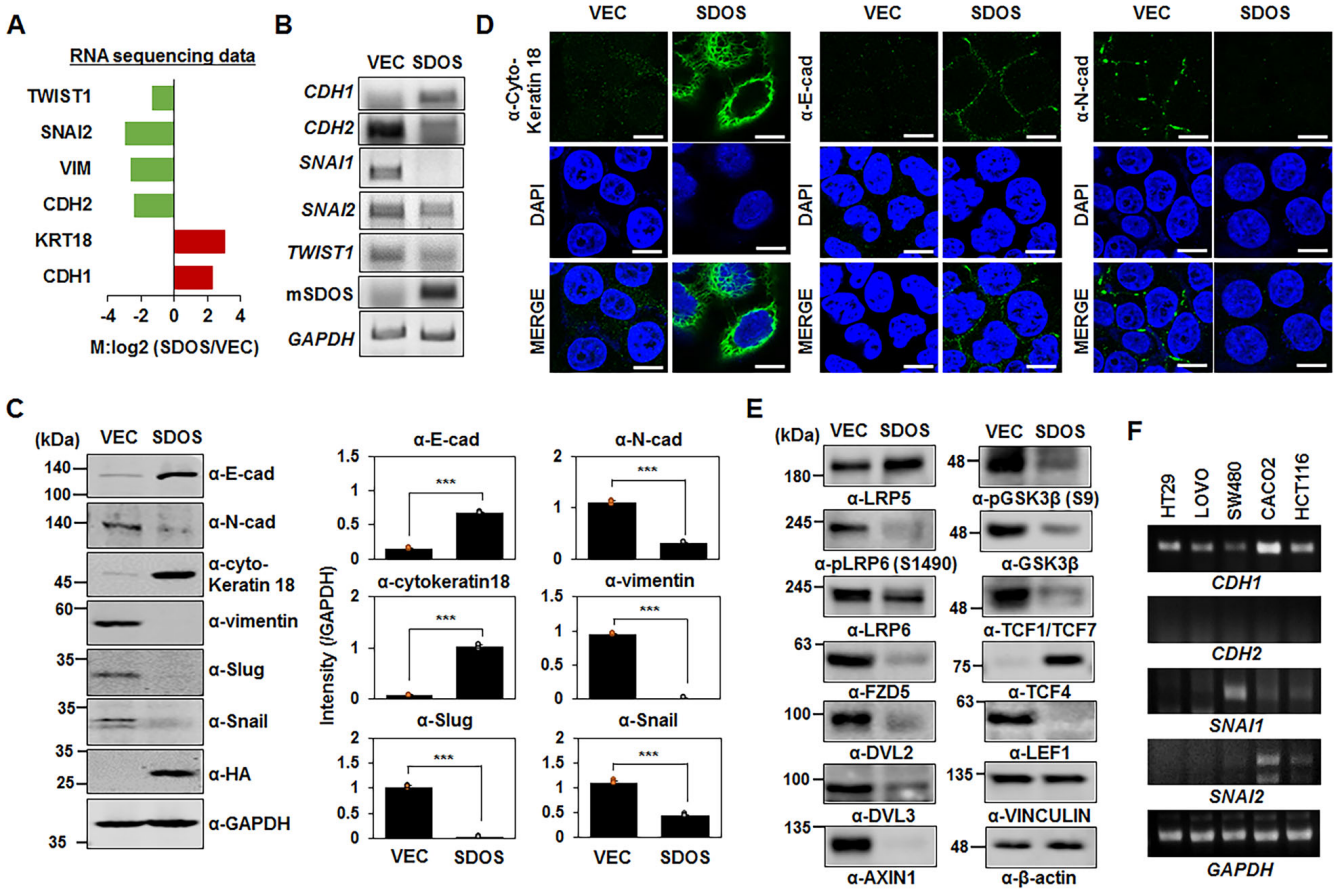
SDOS regulates EMT in colon cancer cells. HCT116 cells were stably transfected with empty vector (VEC) or vector encoding SDOS (SDOS). **A** Expression of EMT-relative genes were analyzed by RNA sequencing. **B** Total RNA was extracted and mRNA levels of EMT markers were determined by RT-PCR. **C** The expression levels of EMT-relative proteins were measured by Western blotting with indicated antibodies (left). GAPDH was used as a loading control. Protein expression levels was quantified (right). **D** Representative confocal immunofluorescence staining images of cytokeratin18 (left), E-cadherin (E-cad, middle) and N-cadherin (N-cad, right) are shown. Nuclei were counterstained with DAPI for visualization. **E** The expression levels of Wnt signaling proteins were measured by immuno blotting with indicated antibodies. β-actin was used as a loading control. Data are presented as mean ± standard deviation (SD). Significant differences between groups are denoted as ^***^p < 0.001. **F** The total RNA extracted from colon cancer cell lines and basal mRNA expression levels of EMT-relative genes were determined by RT-PCR, with GAPDH used as the loading control.

Since the disassembly of epithelial cell-cell contacts is the first step of EMT, we further investigated whether SDOS expression influences EMT (Fig. 6). RNA-seq (Fig. 6A) and RT-PCR (Fig. 6B) analyses revealed that SDOS expression in HCT116 cells increased the mRNA expression level of an epithelial marker (E-cadherin), while decreasing that of a mesenchymal marker (N-cadherin). Consistently, SDOS reduced the mRNA expression levels of snail, slug, and twist, which are transcription factors that downregulate E-cadherin (Fig. 6B). Accordingly, in HCT-SDOS cells, the protein levels of E-cadherin and cytokeratin 18 were increased, but those of N-cadherin, slug, and snail were decreased (Fig. 6C). Immunofluorescence imaging confirmed that HCT-SDOS cells exhibited upregulation of cytokeratin 18 in the cytosol and E-cadherin in the plasma membrane, but downregulation of N-cadherin (Fig. 6D). Given that the Wnt/β-catenin signaling axis is a key regulator of EMT in colon cancer cells [34] and promotes malignant progression [35], we further assessed whether SDOS affects this pathway (Fig. 6E). Consistent with its proposed tumor-suppressive role, SDOS expression resulted in decreased levels of multiple Wnt regulatory proteins. In addition, SDOS reduced the expression of TCF1 and LEF1, both pro-tumorigenic transcription factors [36, 37], while increasing the expression of TCF4, which has been reported to exert tumor-suppressive functions [38]. These findings indicate that SDOS inhibits EMT, likely by restoring epithelial cell architecture. Overall, our data suggest that SDOS helps maintain apical cell–cell adhesion structures in epithelial cells, thereby preventing the acquisition of aggressive traits characteristic of colon cancer cells. Moreover, among the five colon cancer cell lines tested, cells expressing lower levels of SDOS, with the exception of CACO2 cells, exhibited relatively higher expression of mesenchymal markers (Fig. 6F).

### SDOS expression suppresses tumor growth of HCT116 cells in vivo

**Fig. 7:**
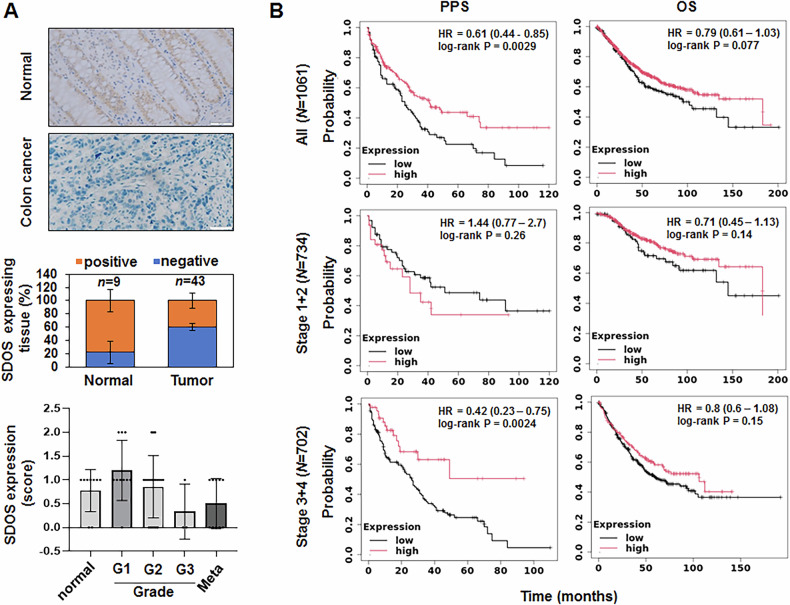
Syndesmos expression is reduced in tissues from patients with advanced colon cancer. **A** Immunohistochemistry analysis was performed to measure the protein level of SDOS in CRC tissue samples and normal tissue samples (top). Magnification, ×400. SDOS-expressing tissue (positive) and non-expressing tissue (negative) were counted and represented with graph (middle). CRC patient divided tumoral grade and counted according to CRC grade (bottom). **B** Kaplan-Meier survival curves showing post-progression survival (PPS) and overall survival (OS) in human colon cancers patients, grouped by high (red) and low (black) SDOS expression levels. Patients were either not selected (all) or selected based on histological tumor stage. The hazard ratio (HR) and log-rank *P*-value are shown in each figure.

Consistent with the antitumorigenic activity of SDOS in HCT116 cells, analysis of colon cancer patient samples showed that SDOS expression was detected in 39.5% of tumor tissue samples compared to 77.8% of adjacent normal tissue samples (Fig. 7A). Notably, grade I (G1, well differentiated) and grade II (G2, moderately differentiated) tissue samples showed SDOS expression levels similar to those of normal tissue, whereas grade III (G3, poorly differentiated) and metastatic (Fig. 7A) tissue samples exhibited significantly lower SDOS expression. In addition, in the Kaplan-Meier (KM) plotter dataset of colon cancer patients, low SDOS expression was significantly associated with poor post-progression survival (PPS). This association was more pronounced in patients with advanced stages (stages 3 and 4) compared to those with early stages (stages 1 and 2) (Fig. 7B). This suggests that, in humans, SDOS expression is negatively correlated with colon carcinogenesis, particularly in the later stages. Interestingly, analysis of the KM plotter dataset for lung cancer patients revealed that low SDOS expression was also significantly associated with poor PPS and overall survival (Supplementary Fig. S3A). Consistent with these clinical observations, transient overexpression of SDOS in the metastatic lung cancer cell line A549 resulted in reduced cell migration (Supplementary Fig. S3B), accompanied by decreased expression of EMT markers (Supplementary Fig. S3C). Collectively, these results suggest that SDOS functions as a tumor suppressor not only in colon cancer but also across multiple carcinoma types.

**Fig. 8:**
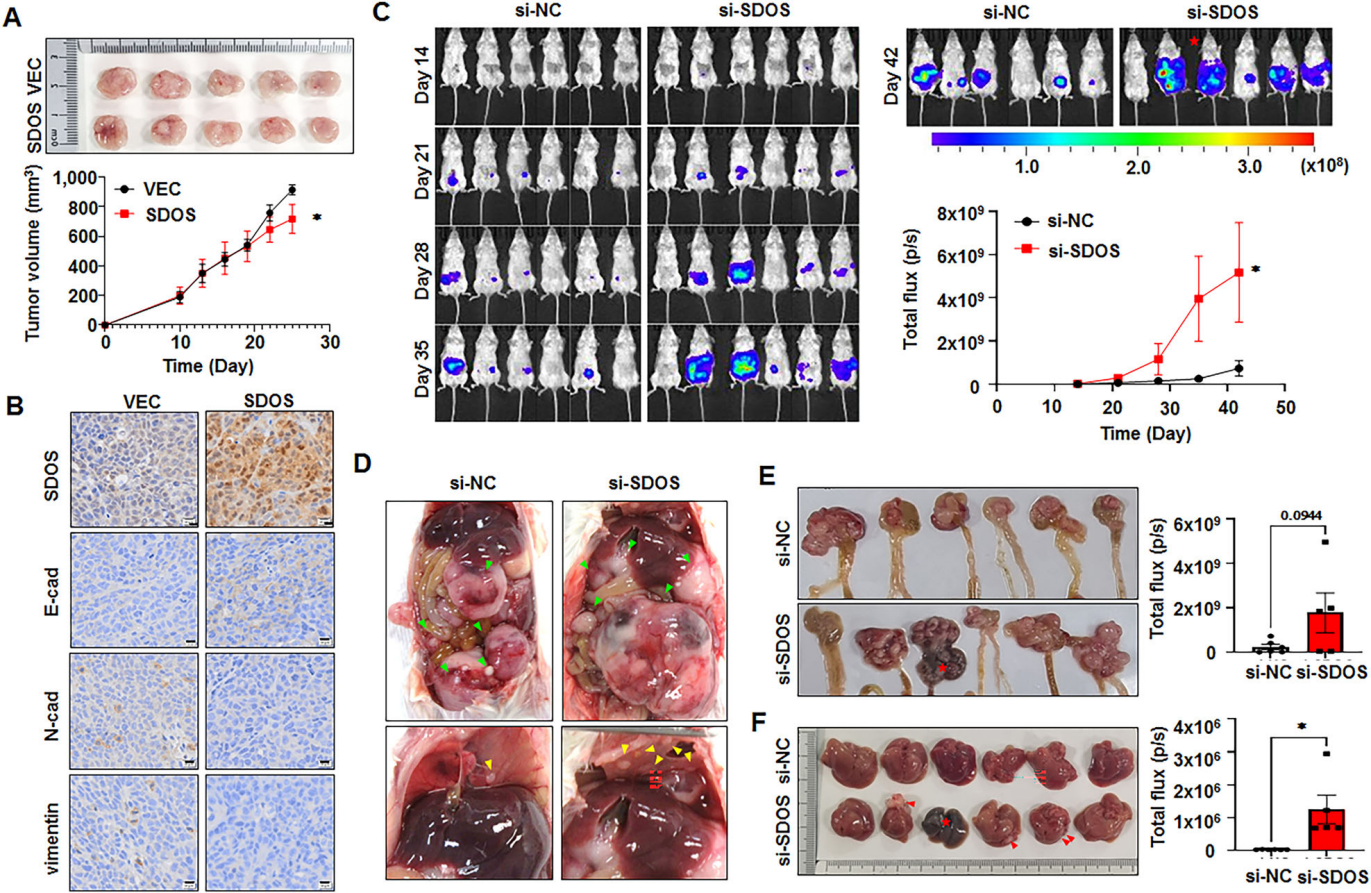
Syndesmos play a role as a tumor suppressor in colon cancer. **A**, **B** Control HCT116 cells (VEC) or HCT-SDOS cells (SDOS) were subcutaneously injected into BALB/c nude mice (*n* = 5 per group). Representative images of tumor are shown (**A**, top). Average tumor volume (mm^3^) at every 3 days post-injection is shown (**A**, bottom). Tumors were subjected to immunohistochemistry anlaysis using indicated antibodies, and representative IHC images are shown (**B**). **C**–**F** CT26-luc cells were transiently transfected with si-NC and si-SDOS and injected into the cecum of BALB/c mice (*n* = 6 per group). Representative in vivo bioluminescence images of tumor development are shown (**C**). The total tumor burden was quantified by photon flux weekly and graphed (**C**, right bottom). The mouse marked with red asterisks died on day 43; therefore, all remaining mice were sacrificed on day 43. Representative images of in vivo metastatic lesions in the intestine, mesentery (green arrow), and diaphragm (yellow arrow) are shown (**D**). Representative ex vivo images of resected cecal tumors (**E**) and liver metastases (F, red arrow) are shown (left). Ex vivo luminescence signals from resected cecal tumors and liver metastases were quantified by photon flux on day 43 and graphed (si-NC, *n* = 6; si-SDOS, *n* = 5, right). Statistical analyses were performed using two-way ANOVA followed by Sidak’s multiple comparisons test (**A**, **C**) and an unpaired Student’s *t* test (**E**, **F**). Data are presented as mean ± standard error of the mean (SEM). Significant differences between groups are denoted as *p < 0.05.

To further investigate the anticancer activity of SDOS in vivo, HCT116 cells overexpressing SDOS (HCT116-SDOS) were injected subcutaneously into BALB/c nude mice and tumor growth was monitored up to 26 days. Tumors derived from HCT116-SDOS cells exhibited a modest reduction in tumor growth compared with those derived from HCT116-VEC cells (Fig. 8A). Immunohistochemical analysis further demonstrated increased E-cadherin and decreased N-cadherin and vimentin expression in HCT116-SDOS-derived tumors relative to HCT116-VEC-derived tumors (Fig. 8B). Furthermore, in an orthotopic colon cancer model generated by cecal injection of CT26-luc cells with siRNA-mediated SDOS knockdown, in vivo bioluminescence imaging revealed significantly increased tumor burden in the si-SDOS group (5.18 × 10^9^ p/s) compared with the si-NC group (7.40 × 10^8^ p/s) on day 42 (Fig. 8C). Consistent with these findings, the si-SDOS group exhibited enhanced primary cecal tumors, as demonstrated by macroscopic examination and increased ex vivo photon flux in the cecal tumors (si-NC: 2.39 × 10^8^, si-SDOS: 1.78 × 10^9^ p/s, Fig. 8D, E). In addition, increased metastatic lesions were detected in the intestine and mesentery (green arrow), diaphragm (yellow arrow), and liver (red arrow) in the si-SDOS group (Fig. 8D). Ex vivo bioluminescence analysis further demonstrated a significant increase in liver metastatic burden in the si-SDOS group (1.25 × 10^6^ p/s) compared with the si-NC group (2.93 × 10^4^ p/s) (Fig. 8E). Together, these in vivo results suggest that SDOS may suppress colon cancer development and metastasis.

## Discussion

Although SDOS is known to regulate actin cytoskeletal formation during normal cell function, the relationship between SDOS-mediated cytoskeletal organization and its role as a tumor suppressor had not previously been investigated. Here, we show for the first time that SDOS expression contributes to the formation of stable cell-cell and cell-ECM interactions and thereby helps maintain epithelial cell architecture. In HCT116 colon cancer cells, SDOS overexpression shifted the mesenchymal morphology to an epithelial morphology (Fig. 3A) and enhanced the formation of cell-ECM junctions, including focal adhesions (Fig. 3D, E), and cell-cell junctions, including adherens junctions (Figs. 4 and 5). Consistently, our RNA-seq data showed that SDOS upregulated components of adherens junctions, tight junctions, and focal adhesions (Fig. 2B). Therefore, SDOS expression architecturally converted the cancer cell morphology to a normal epithelial cell morphology in colon carcinoma cells (i.e., mesenchymal-to-epithelial transition, or MET). Consistently, SDOS overexpression in HCT116 cells downregulated mesenchymal markers (N-cadherin, slug, snail, vimentin and twist) and upregulated epithelial markers (E-cadherin and cytokeratin 18) (Fig. 6). Previous studies showed that SDOS promotes the association of paxillin with syndecan-4 to regulate actin stress fiber assembly in fibroblasts [3, 4]. SDOS was also shown to regulate paxillin distribution and focal adhesion formation to promote the laminar flow-induced cytoskeletal reorganization of endothelial cells [39]. Therefore, SDOS appears to play a general role in regulating cell architecture across different cell types.

Consistent with the impacts of SDOS on epithelial cell morphology, SDOS expression was lower in highly metastatic colon cancer cells compared to weakly metastatic colon cancer cells (Fig. 1). Additionally, SDOS expression was lower in colon cancer tissue samples compared to adjacent normal tissues (Fig. 7A), and lower SDOS expression was significantly associated with poor post-progression survival, particularly in the later stages (Fig. 7B). These findings suggest that SDOS expression is negatively correlated with colon carcinogenesis in humans. Indeed, exogenous expression of SDOS decreased the EMT, migratory activity, and proliferation of HCT116 cells, whereas siRNA-mediated knockdown of SDOS increased the migration and growth of HT29 cells (Fig. 1). Consistent with these findings, exogenous SDOS expression modestly reduced tumor growth (Fig. 8A) and decreased the EMT (Fig. 8B) in HCT116 xenografts. Furthermore, in the orthotopic mouse model, silencing SDOS in CT26-luc cells enhanced the tumor burden in the cecum and promoted metastatic dissemination to the intestine, mesentery, diaphragm, and liver (Fig. 8C–E), supporting the notion that the SDOS-mediated regulation of cellular architecture could play a role in tumor suppression.

Interestingly, we found that SDOS contributes to regulating all types of cell adhesion junctions in epithelial cells. Considering the relatively small size of SDOS, it is unlikely to function as a scaffolding protein to connect all intracellular components of cell junctions. However, our data revealed that SDOS interacted with both E-cadherin and β-catenin in HCT-SDOS cells (Figs. 4 and 5) and HT-29 cells (Supplementary Fig. S1), suggesting that SDOS may directly contribute to adherens junction formation through E-cadherin. Since adherens junction formation is a prerequisite for tight junction assembly [31, 32, 40], our findings indicate that SDOS is likely to indirectly mediate tight junction formation. Consistent with this, we found that SDOS in the basal aspect of HCT116 cells interacted with syndecan-4 (Fig. 3), which regulates the formation of focal adhesions. Therefore, SDOS interacts with various molecules to regulate epithelial cell junctions as an intracellular component. SDOS also plays diverse functional roles in different subcellular locations, in addition to the plasma membrane: In the nucleus, SDOS appears to critically regulate the DNA double-strand break repair pathway by regulating the recruitment of 53BP1 [41–44]. In the cytoplasm, SDOS interacts with the molecular chaperone, TRAP1, at the endoplasmic reticulum to regulate primary cilia formation-related mRNA translation [45]. However, it is not yet clear how SDOS regulates various functions across different subcellular locations and cell types. However, the upstream regulatory mechanisms responsible for SDOS downregulation remain unclear. Because epithelial–mesenchymal plasticity and cell adhesion are regulated by complex and multifactorial signaling networks, future studies will be required to systematically investigate the pathways that control SDOS expression, including potential contributions from Wnt signaling and EMT-associated transcription factors.

Despite these findings, it remains unclear how SDOS coordinates its diverse functions across different subcellular locations and cell types. Moreover, the upstream regulatory mechanisms responsible for SDOS downregulation are still poorly understood. Because epithelial-mesenchymal plasticity and cell adhesion are governed by complex and multifactorial signaling networks, future studies will be required to systematically investigate the pathways controlling SDOS expression, including the potential involvement of Wnt signaling and EMT-associated transcription factors.

In summary, our present work shows for the first time that SDOS plays dual roles in regulating epithelial cell architecture. At the basal cell layer, SDOS induces stable cell-ECM interaction through syndecan-4. Simultaneously, in the lateral layers, SDOS regulates the organization of adherens junctions through regulating E-cadherin and β-catenin interactions. This creates cell polarity, a typical morphology for epithelial cells, which stabilizes epithelial cell architecture and increases resistance to the development of a mesenchymal cell phenotype and carcinomas. Conversely, the loss of SDOS in epithelial cells leads colon cancer cells to acquire tumor growth and metastatic properties. Although the regulatory role of SDOS in other carcinomas remains unknown, our data clearly demonstrate that it plays an antitumor role in epithelial cells derived from colonic tumors. This represents a novel regulatory role for SDOS in epithelial cells in the context of colon carcinogenesis.

## Supplementary information

Revised Supplementary information

Original Western blot

## Data Availability

All other raw data are available upon request from the corresponding author.
