# Supplementary material for: Syndesmos functions as a tumor suppressor by facilitating epithelial cell adhesion mediated by interactions of E-cadherin and β-catenin
Source: Cell Death Dis. 2026 May 19;17(1):633. doi: 10.1038/s41419-026-08857-0 (PMC13351065; doi:10.1038/s41419-026-08857-0)
Supplement: Supplementary file 1 — Revised Supplementary information [file 41419_2026_8857_MOESM1_ESM.docx]

**Supplementary Information**

**Syndesmos functions as a tumor suppressor by facilitating epithelial cell adhesion mediated by interactions of E-cadherin and β-catenin**

Jisun Hwang^1*^, Ga-Eun Lim^2*^, Bohee Jang^1^, Jee Young Sung^2^, Hyewon Shim^3^, Hyeryun Kwon^3^, Eun Kyung Hong^4^, Eek-hoon Jho^3^, Yong-Nyun Kim^2@^, Eok-Soo Oh^1@^

**^@^**To whom correspondence may be addressed: **Eok-Soo Oh**, Department of Life Sciences, Ewha Womans University, 52, Ewhayeodae-gil, Seodaemoon-Gu, Seoul 120-750 Korea, Tel.: (82)-2-3277-3761; Fax: (82)-2-3277-3760; E-mail: OhES@ewha.ac.kr, **Yong-Nyun Kim** Cancer Metastasis Branch, Division of Cancer Biology, National Cancer Center, 323 Ilsan-ro, Ilsandong-gu, Gyeonggi-do, Goyang-si 10408, Korea, Tel.: (82)-31-920-2415; Fax: (82)-31-920-2468; E-mail: ynk@ncc.re.kr

**This supplementary information includes:**

Supplementary Figures S1, S2, S3, and S4

Supplementary Figure legend

**Supplementary Figure and Figure legends**

**
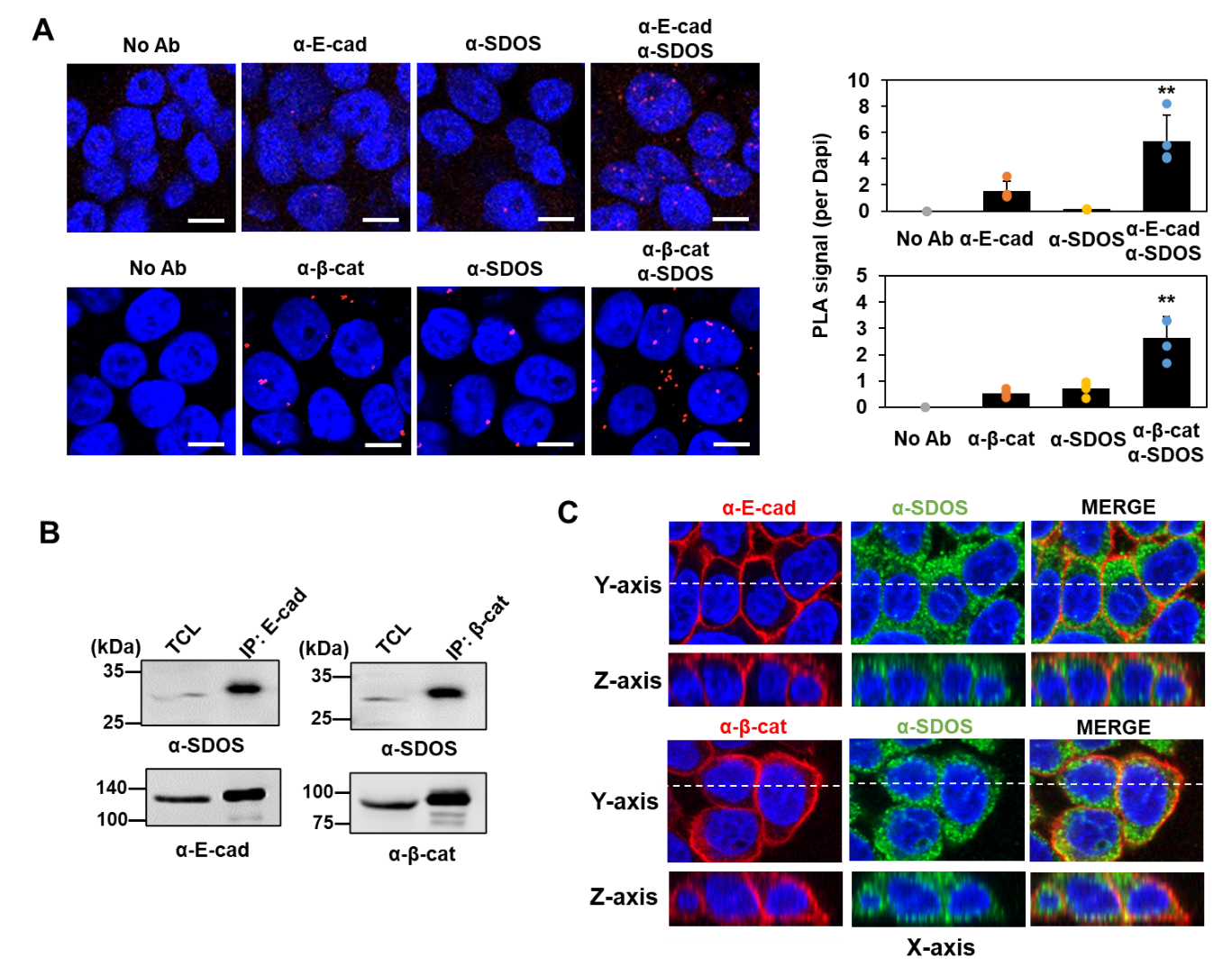
**

**Supplementary figure 1. SDOS interacts with E-cadherin and β-catenin at the cell surface of HT29 cells**. (A) The *in situ* proximity ligation assay using HT29 cells was performed with anti-E-cadherin (E-cad) and anti-SDOS antibodies. Images of confocal immunofluorescence staining are shown. The nuclei were counterstained with DAPI (left). The PLA signals in the field were shown graphically (right). Data are presented as mean ± standard deviation (SD). Significant differences between groups are denoted as ^**^*p* <0.01. (B) HT29 total cell lysates (TCL) were immunoprecipitated with either anti-E-cad or -β-catenin (β-cat) antibodies and immunoblotted with the indicated antibodies. (C) HT29 cells plated on the coverslip were probed with either anti-E-cad antibody (red) and anti-SDOS antibody (green, top) or anti-β-cat antibody (red) and anti-SDOS antibody (green, bottom). Representative images of confocal immunofluorescence staining are shown. The nuclei were counterstained with DAPI.

**
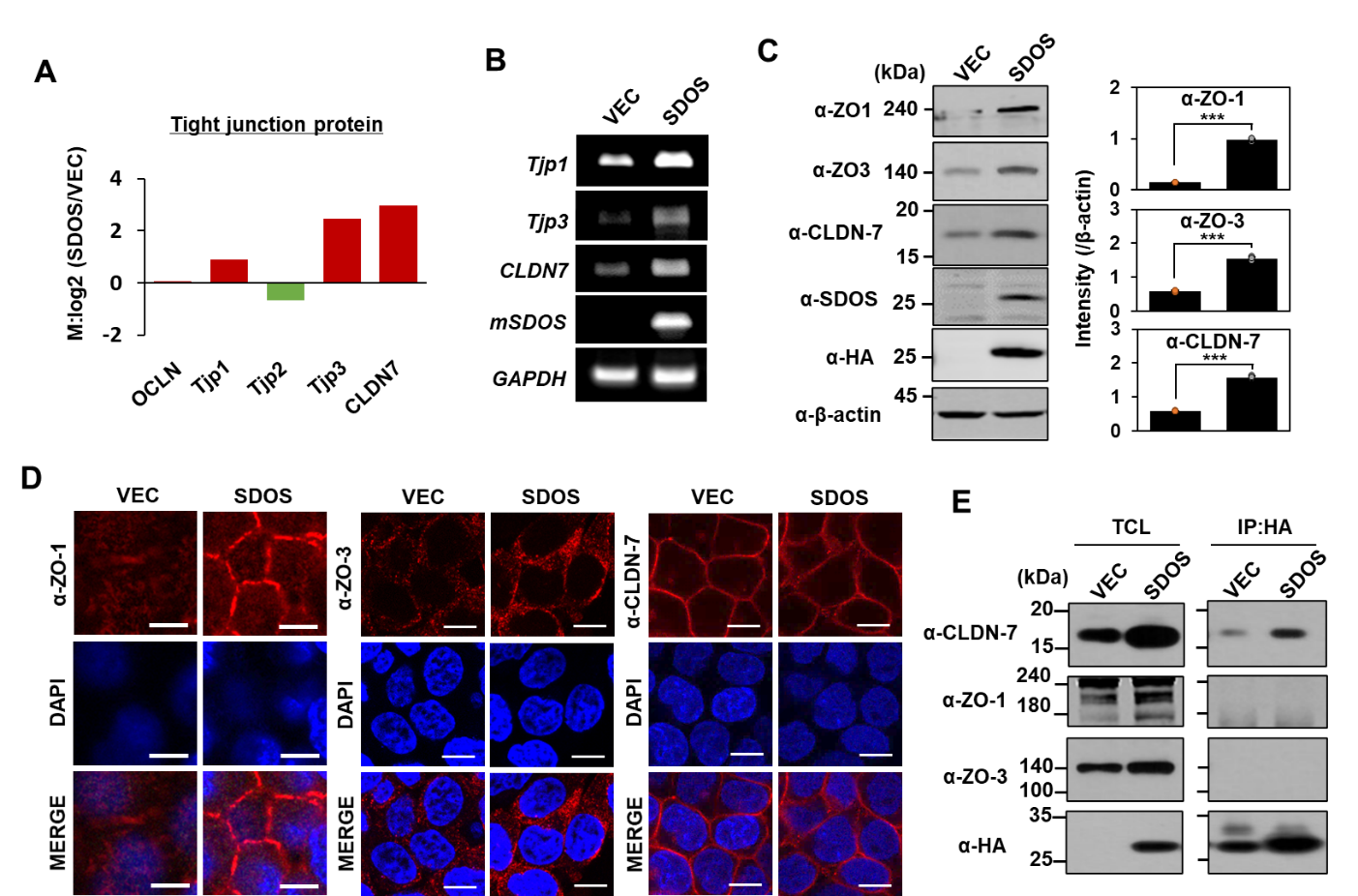
Supplementary figure 2. SDOS expression enhances tight junction formation in HCT116 cells.** HCT116 cells were stably transfected with empty vector (VEC) or vector encoding SDOS (SDOS). (A) Expression of tight junction protein genes were analyzed by RNA sequencing. (B) Total RNA was extracted and mRNA levels of tight junction protein genes were determined by RT-PCR. (C) Cell lysates were subjected to Western blotting with the indicated antibodies (left). Protein expression levels was quantified (right). Data are presented as mean ± standard deviation (SD). Significant differences between groups are denoted as ^***^*p* <0.001. (D) Representative confocal immunofluorescence staining images of ZO-1 (left) and ZO-3 (right) are shown. Nuclei were counterstained with DAPI for visualization. (E) Total cell lysates (TCL) were immunoprecipitated with anti-HA antibody and immunoblotted with the indicated antibodies.

**
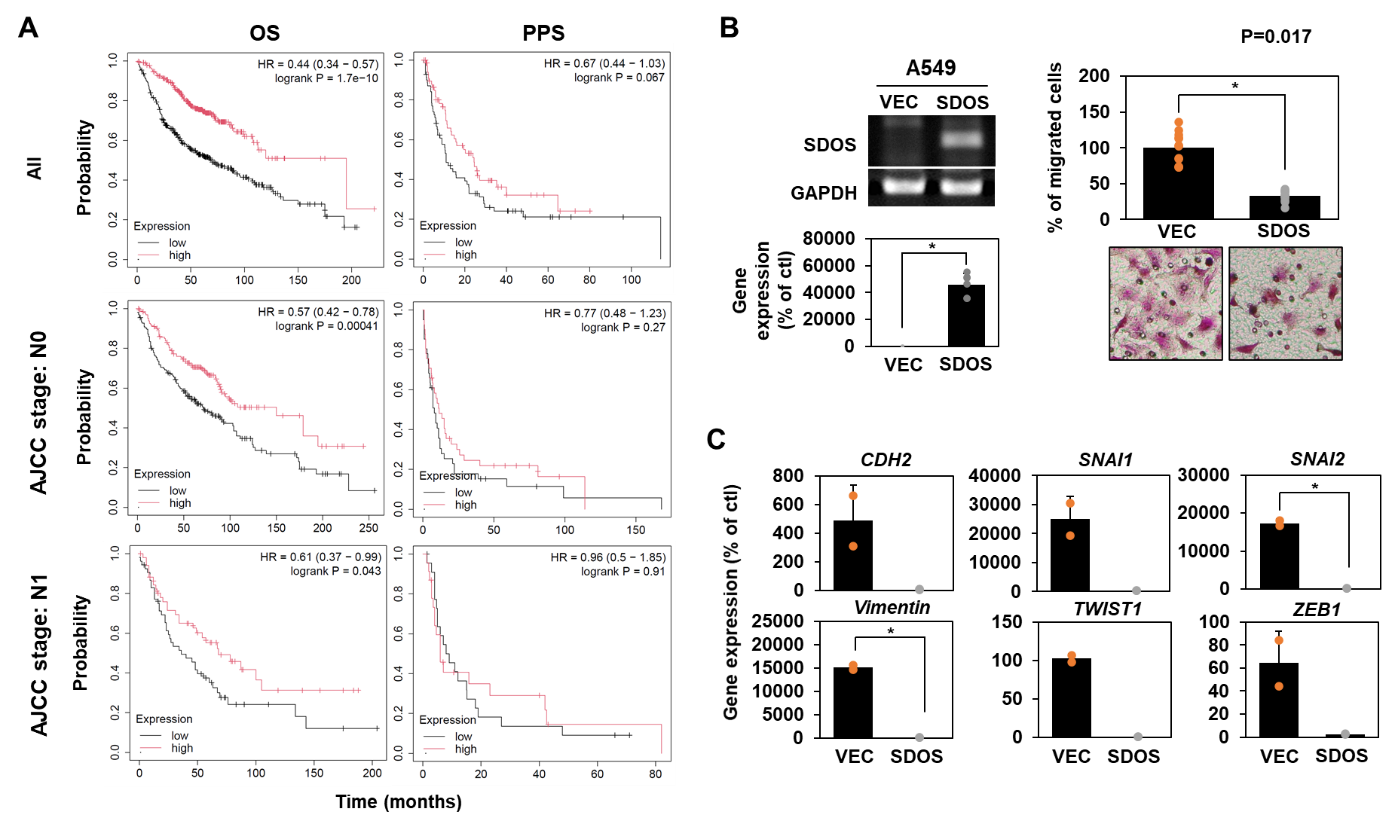
**

**Supplementary figure 3. SDOS expression reduces cell migration in A549 lung cancer cells.** (A) Kaplan-Meier survival curves showing overall survival (OS) and post-progression survival (PPS) in human lung cancer-adenocarcinoma patients, grouped by high (red) and low (black) SDOS expression levels. Patients were either not selected (all) or selected based on histological tumor stage. The hazard ratio (HR) and log-rank P-value are shown in each figure. (B, C) A549 lung cancer cells were transiently transfected with empty vector (VEC) or vector encoding SDOS (SDOS). (B) The mRNA expression levels of SDOS were analyzed by RT-PCR and quantified. GAPDH was used as a loading control (left). Transwell migration assay were performed as described in the Materials and Methods (right). (C) mRNA expression levels of EMT-relative genes by quantitative real-time PCR analysis. The related transcript level was normalized to the GAPDH level. Data are presented as mean ± standard deviation (SD). Significant differences between groups are denoted as ^*^*p* <0.05.


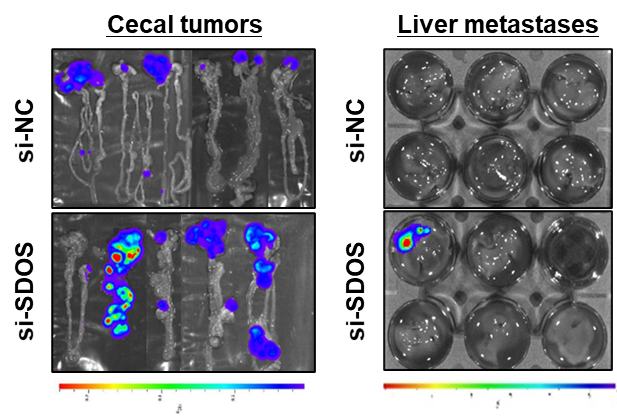


**Supplementary figure 4. *Ex vivo* bioluminescence imaging of cecal tumors and liver metastases.** Representative *ex vivo* IVIS bioluminescence images of resected cecal tumors (left) and liver metastases (right) from BALB/c mice injected with CT26-luc cells transiently transfected with si-NC, or si-SDOS. On day 43, mice were administered luciferin, sacrificed, and subjected to *ex vivo* IVIS imaging of resected cecal tumors and livers. Pseudocolor scale indicates photon flux intensity.
