# Supplementary material for: Syndesmos functions as a tumor suppressor by facilitating epithelial cell adhesion mediated by interactions of E-cadherin and β-catenin
Source: Cell Death Dis. 2026 May 19;17(1):633. doi: 10.1038/s41419-026-08857-0 (PMC13351065; doi:10.1038/s41419-026-08857-0)
Supplement: Supplementary file 2 — Original Western blot [file 41419_2026_8857_MOESM2_ESM.pdf]

Fig. 3B

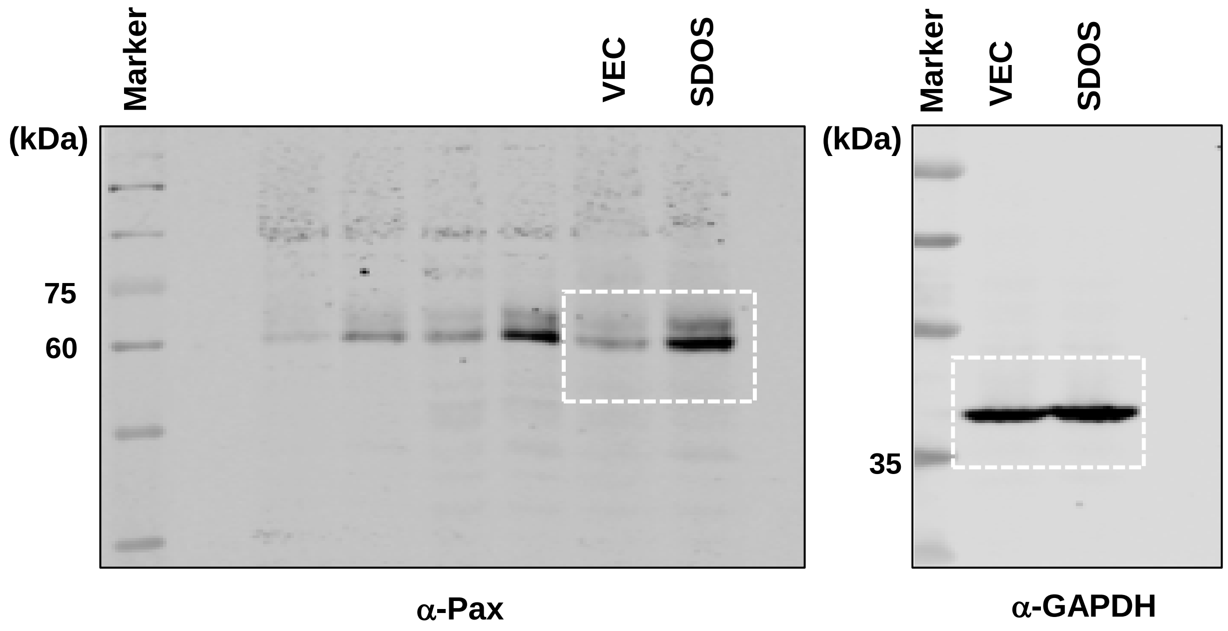

Fig. 4 C

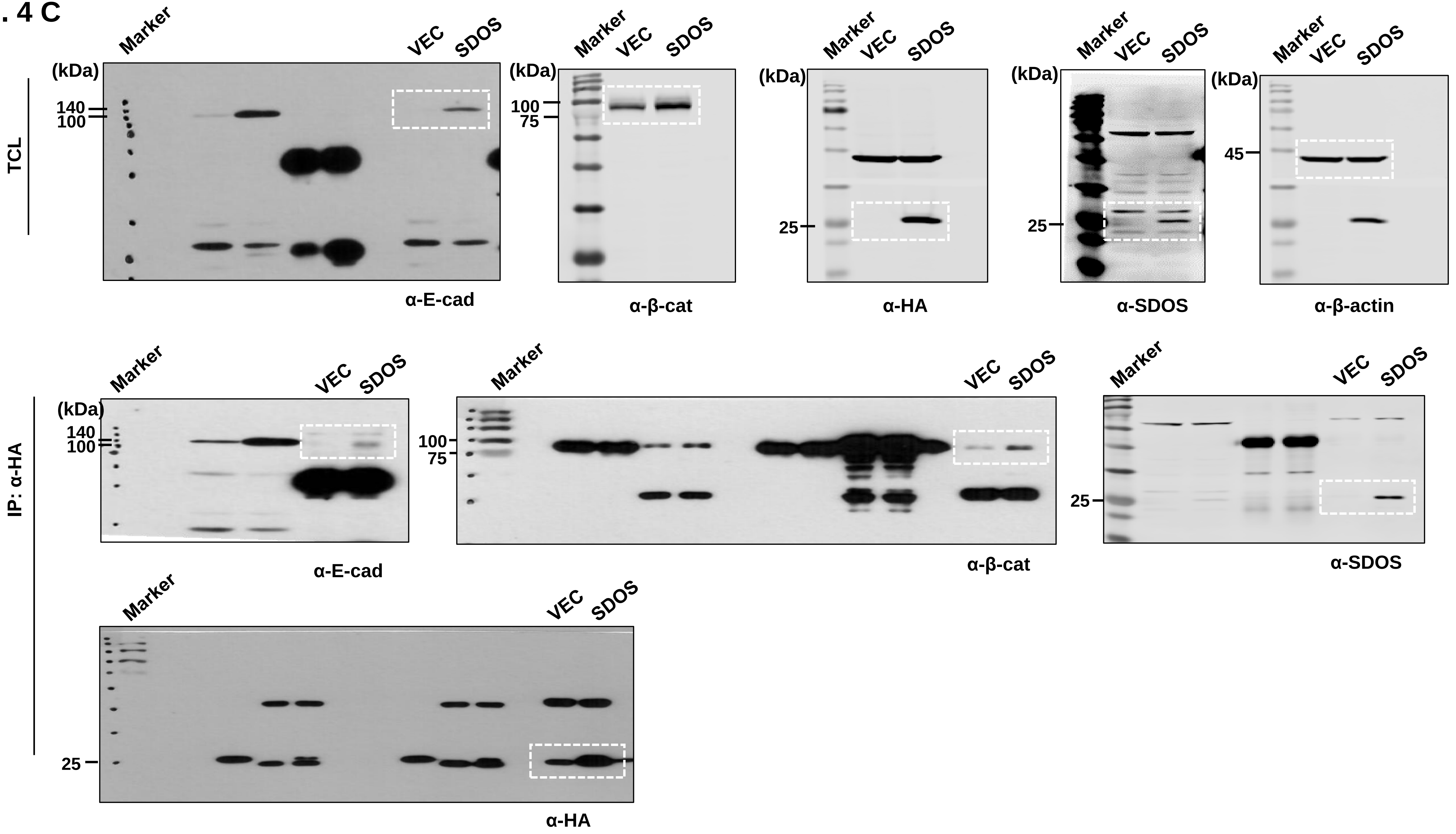
TCL
Marker
VEC
SDOS
(kDa)
140
100
75
25
45
α-E-cad
α-β-cat
α-HA
α-SDOS
α-β-actin
IP: α-HA

Fig. 4 C

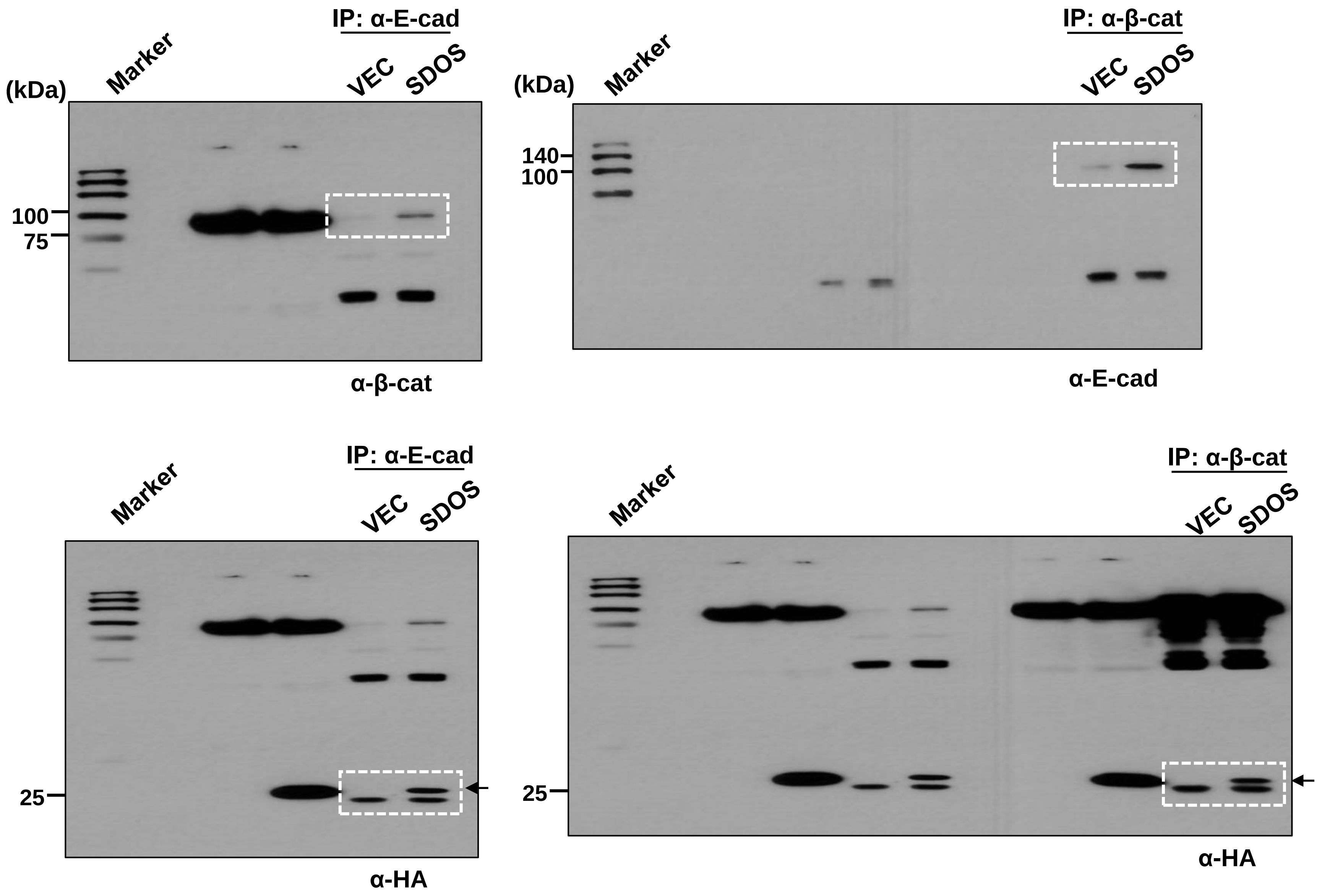
IP: α-E-cad
Marker
VEC
SDOS
(kDa)
100
75
α-β-cat
IP: α-β-cat
Marker
VEC
SDOS
(kDa)
140
100
α-E-cad
IP: α-E-cad
Marker
VEC
SDOS
25
α-HA
IP: α-β-cat
Marker
VEC
SDOS
25
α-HA

Fig. 6C

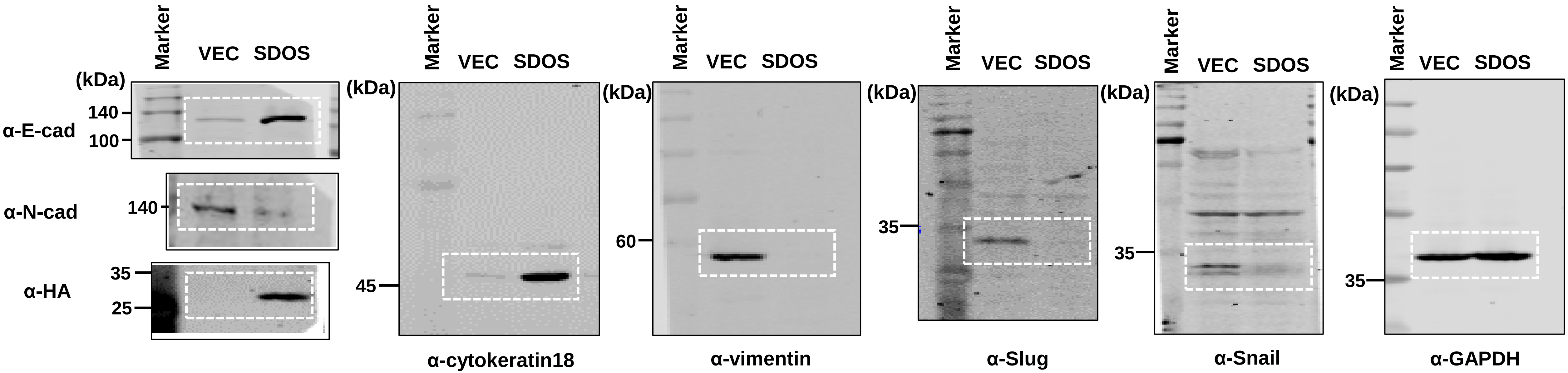
Marker
VEC
SDOS
(kDa)
140
100
α-E-cad
α-N-cad
140
α-HA
35
25
(kDa)
45
α-cytokeratin18
(kDa)
60
α-vimentin
(kDa)
35
α-Slug
(kDa)
35
α-Snail
(kDa)
35
α-GAPDH

Fig. 6E

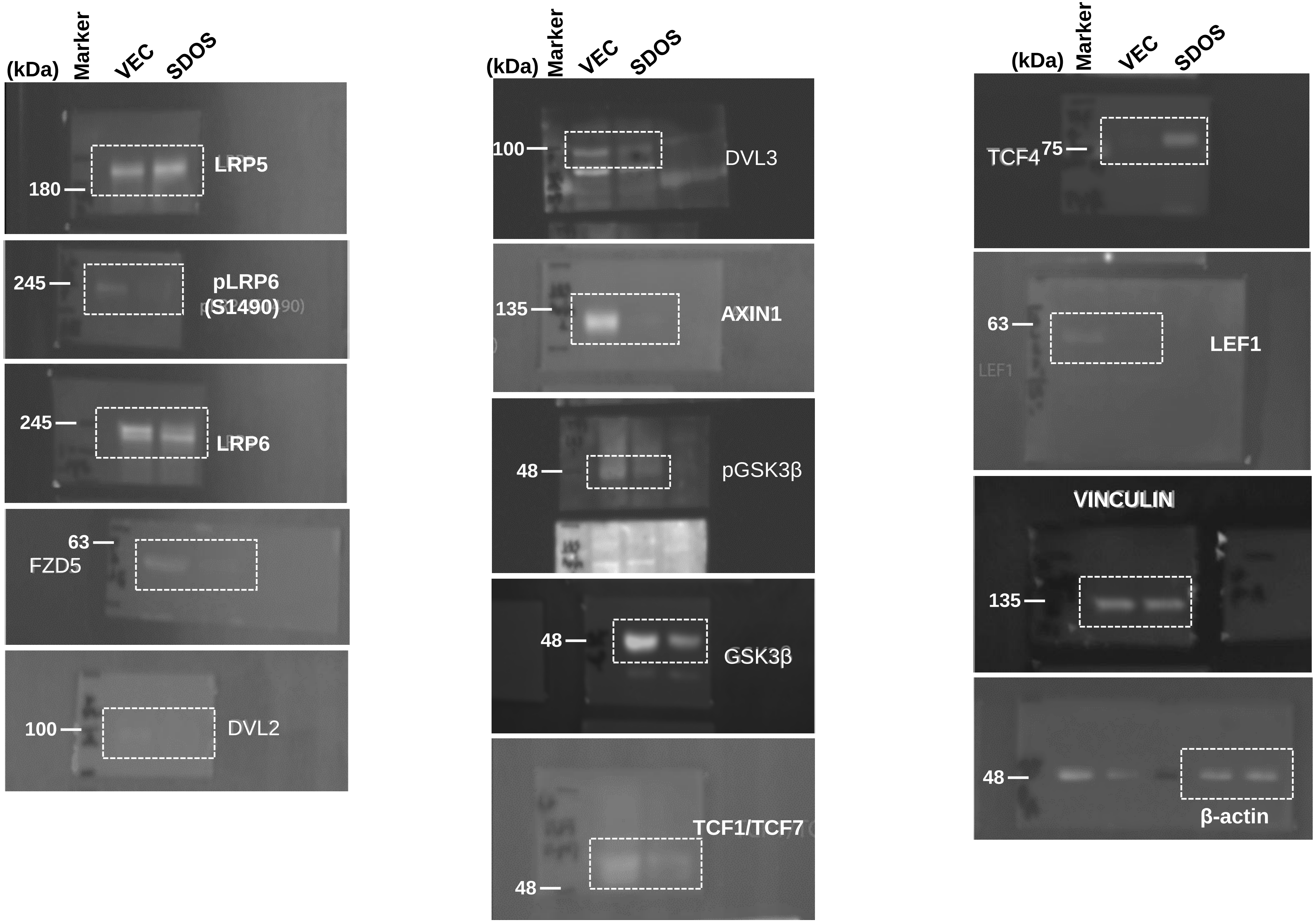

(kDa)
Marker
VEC
SDOS
LRP5
180
pLRP6
(S1490)
245
LRP6
245
63
FZD5
100
DVL2
DVL3
100
AXIN1
135
pGSK3β
48
GSK3β
48
TCF1/TCF7
48
TCF4
75
LEF1
63
VINCULIN
135
48
β-actin

Fig. S1B

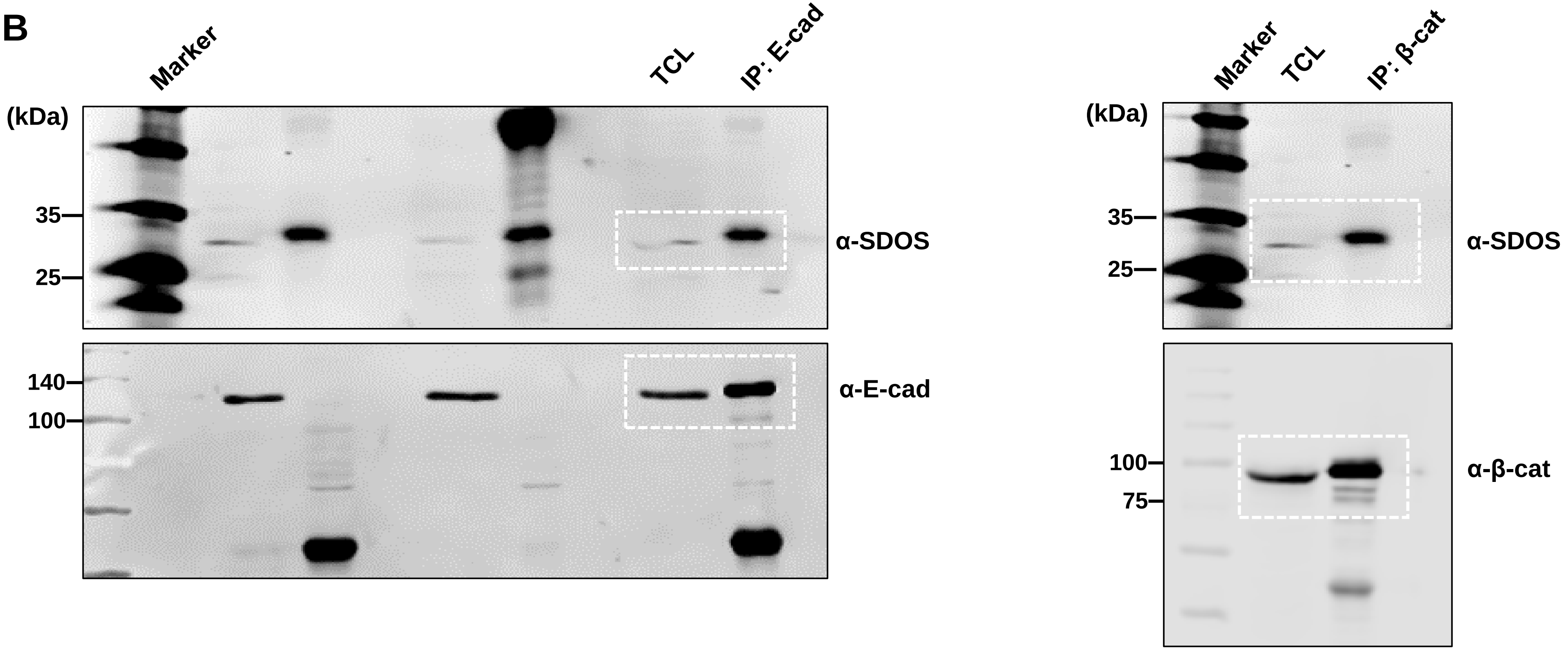
B
Marker
TCL
IP: E-cad
(kDa)
35
25
α-SDOS
140
100
α-E-cad
Marker
TCL
IP: β-cat
(kDa)
35
25
α-SDOS
100
75
α-β-cat

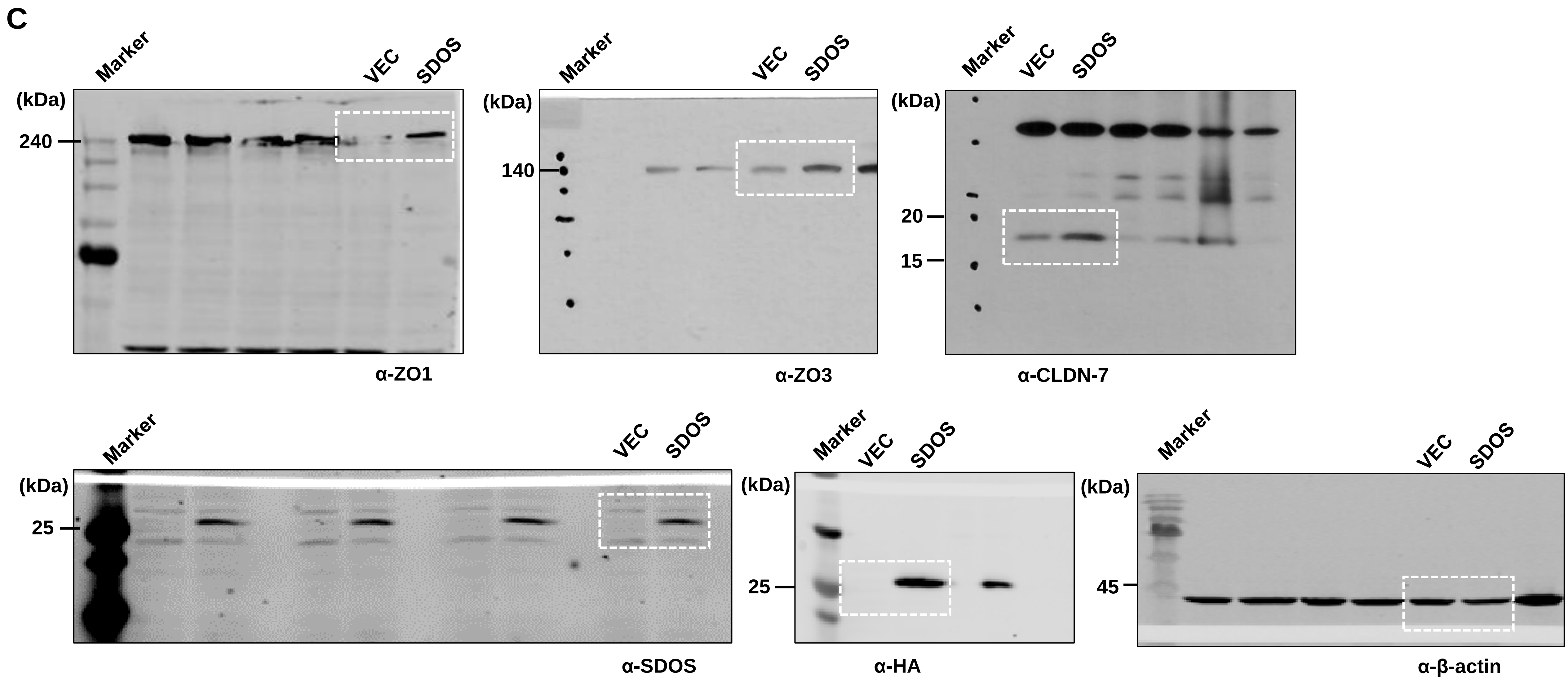
C
Marker
VEC
SDOS
(kDa)
240
α-ZO1
Marker
VEC
SDOS
(kDa)
140
α-ZO3
Marker
VEC
SDOS
(kDa)
20
15
α-CLDN-7
Marker
VEC
SDOS
(kDa)
25
α-SDOS
Marker
VEC
SDOS
(kDa)
25
α-HA
Marker
VEC
SDOS
(kDa)
45
α-β-actin

Fig. S2E

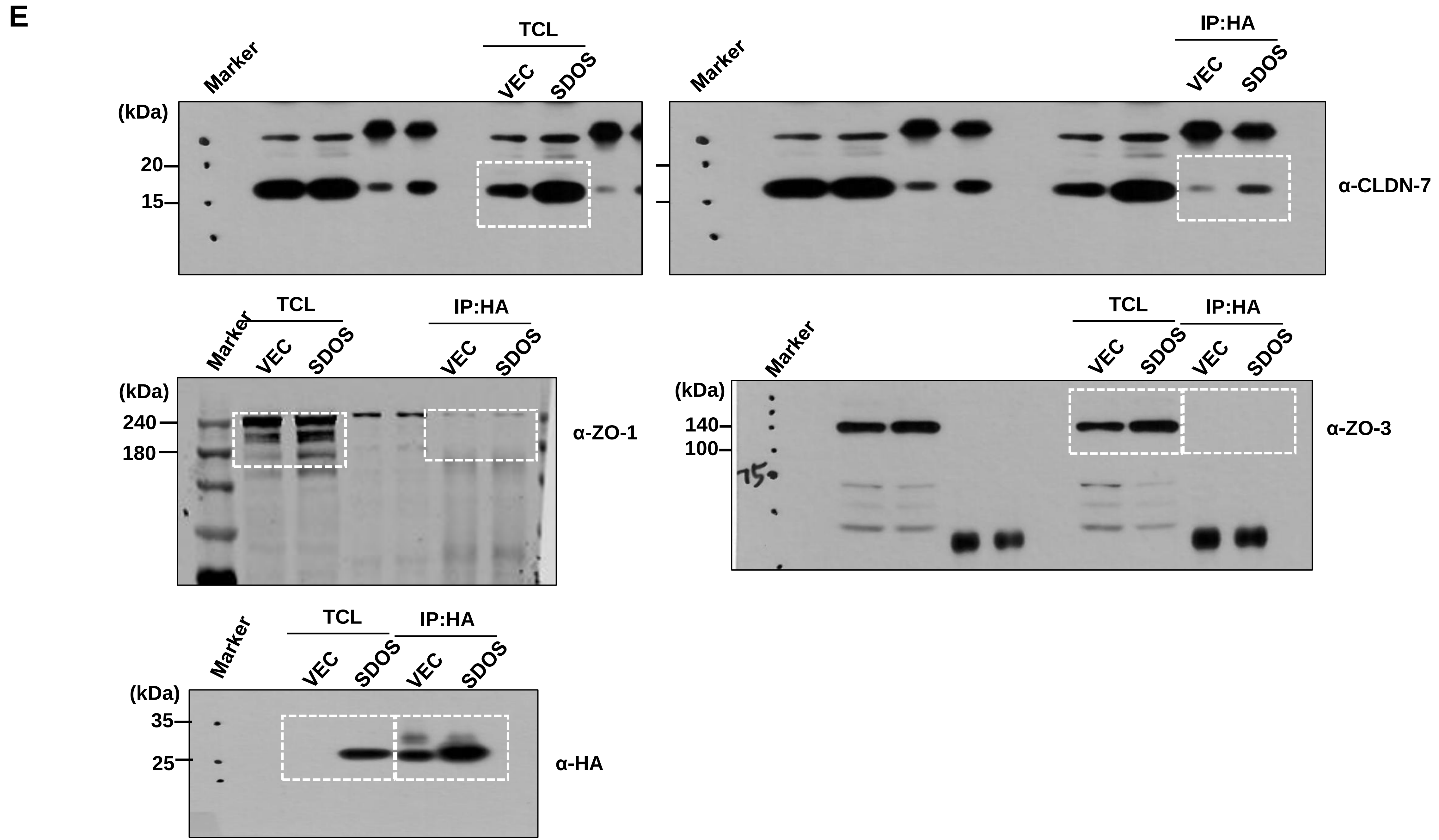
E
TCL
IP:HA
Marker
VEC
SDOS
(kDa)
20
15
α-CLDN-7
240
180
α-ZO-1
140
100
75
α-ZO-3
35
25
α-HA
